# Spatial atlas of the mouse central nervous system at molecular resolution

**DOI:** 10.1038/s41586-023-06569-5

**Published:** 2023-09-27

**Authors:** Hailing Shi, Yichun He, Yiming Zhou, Jiahao Huang, Kamal Maher, Brandon Wang, Zefang Tang, Shuchen Luo, Peng Tan, Morgan Wu, Zuwan Lin, Jingyi Ren, Yaman Thapa, Xin Tang, Ken Y. Chan, Benjamin E. Deverman, Hao Shen, Albert Liu, Jia Liu, Xiao Wang

**Affiliations:** 1 Broad Institute of MIT and Harvard, Cambridge, MA USA; 2 Department of Chemistry, Massachusetts Institute of Technology, Cambridge, MA USA; 3 John A. Paulson School of Engineering and Applied Sciences, Harvard University, Boston, MA USA; 4 Computational and Systems Biology PhD Program, Massachusetts Institute of Technology, Cambridge, MA USA; 5 Department of Electrical Engineering and Computer Science, Massachusetts Institute of Technology, Cambridge, MA USA; 6 Department of Biology, Massachusetts Institute of Technology, Cambridge, MA USA; 7 Klarman Cell Observatory, Broad Institute of MIT and Harvard, Cambridge, MA USA; 8 Department of Chemistry and Chemical Biology, Harvard University, Cambridge, MA USA; 9 Stanley Center for Psychiatric Research, Broad Institute of MIT and Harvard, Cambridge, MA USA

**Keywords:** RNA sequencing, Molecular neuroscience, Transcriptomics, Transcriptomics, Genetic vectors

## Abstract

Spatially charting molecular cell types at single-cell resolution across the 3D volume is critical for illustrating the molecular basis of brain anatomy and functions. Single-cell RNA sequencing has profiled molecular cell types in the mouse brain^1,2^, but cannot capture their spatial organization. Here we used an in situ sequencing method, STARmap PLUS^3,4^, to profile 1,022 genes in 3D at a voxel size of 194 × 194 × 345 nm^3^, mapping 1.09 million high-quality cells across the adult mouse brain and spinal cord. We developed computational pipelines to segment, cluster and annotate 230 molecular cell types by single-cell gene expression and 106 molecular tissue regions by spatial niche gene expression. Joint analysis of molecular cell types and molecular tissue regions enabled a systematic molecular spatial cell-type nomenclature and identification of tissue architectures that were undefined in established brain anatomy. To create a transcriptome-wide spatial atlas, we integrated STARmap PLUS measurements with a published single-cell RNA-sequencing atlas^1^, imputing single-cell expression profiles of 11,844 genes. Finally, we delineated viral tropisms of a brain-wide transgene delivery tool, AAV-PHP.eB^5,6^. Together, this annotated dataset provides a single-cell resource that integrates the molecular spatial atlas, brain anatomy and the accessibility to genetic manipulation of the mammalian central nervous system.

## Abstract (web summary)

In situ spatial transcriptomic analysis of more than 1 million cells are used to create a 200-nm-resolution spatial molecular atlas of the adult mouse central nervous system and identify previously unknown tissue architectures.

## Main

Deciphering spatial arrangements of molecularly defined cell types (hereafter referred to as molecular cell types) at single-cell resolution in the nervous system is fundamental for understanding the molecular architecture of its anatomy, function and disorders. Although single-cell RNA sequencing (scRNA-seq) has revealed the complexity and diversity of cell-type composition in the mouse brain^1,2^, it provides little to no spatial information. Emerging spatial transcriptomic methods have shed light on the molecular organization of mouse brains^7^. However, existing datasets either have limited spatial resolution^8^ (100 µm)—hindering bona fide single-cell analysis—or are restricted to particular brain subregions^9–11^. Therefore, a single-cell-resolved spatial atlas across the entire central nervous system (CNS) would be highly desirable to fully unveil molecular cell types and tissue architectures.

**Fig. 1:**
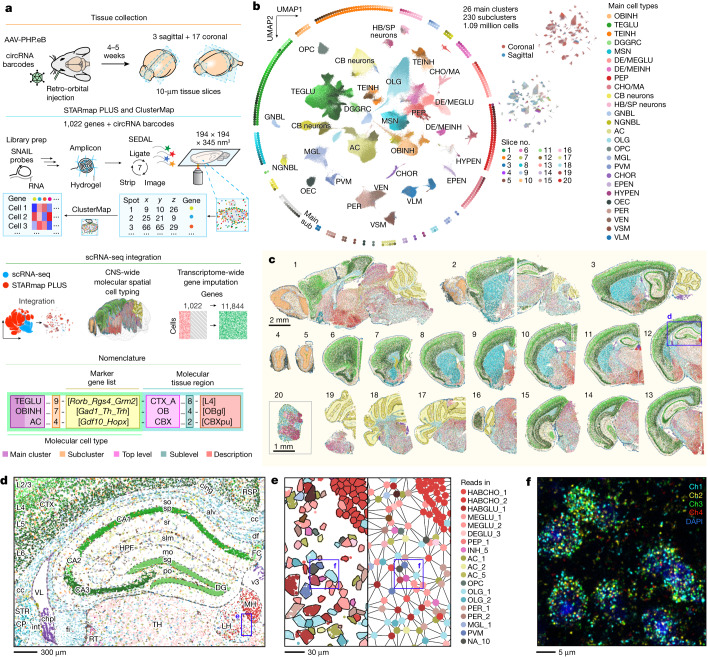
Spatial maps of molecular cell types across the adult mouse CNS at subcellular resolution. **a**, Overview of the study. Mouse brain tissue slices were collected four to five weeks after systemic administration of barcoded AAVs. STARmap PLUS^3,4^ was performed to detect single RNA molecules from a targeted list of 1,022 endogenous genes and the trans-expressed AAV barcodes. The RNA spot matrix was converted to a cell-by-gene expression matrix via ClusterMap^12^. By integrating with existing mouse CNS scRNA-seq data^1^, we generated a CNS spatial atlas with cell cluster nomenclatures jointly defined by molecular cell types and molecular tissue regions, and imputed single-cell transcriptome-wide expression profiles. circRNA, circular RNA; AC, astrocytes; CB, cerebellum; CHO/MA, cholinergic and monoaminergic neurons; CHOR, choroid plexus epithelial cells; DE/MEGLU, diencephalon/mesencephalon excitatory neurons; DE/MEINH, diencephalon/mesencephalon inhibitory neurons; DGGRC, dentate gyrus granule cells; EPEN, ependymal cells; GNBL, glutamatergic neuroblasts; HB/SP, hindbrain/spinal cord; HYPEN, subcommissural organ hypendymal cells; MGL, microglia; MSN, telencephalon projecting inhibitory neurons (or medium spiny neurons); NGNBL, non-glutamatergic neuroblasts; OBINH, olfactory inhibitory neurons; OEC, olfactory ensheathing cells; OLG, oligodendrocytes; OPC, oligodendrocyte precursor cells; PEP, peptidergic neurons; PER, pericytes; PVM, perivascular macrophages; TEGLU, telencephalon projecting excitatory neurons; TEINH, telencephalon inhibitory interneurons; VEN, vascular endothelial cells; VLM, vascular and leptomeningeal cells; VSM, vascular smooth muscle cells. **b**, Uniform manifold approximation and projection^50^ (UMAP) of 1.09 million cells coloured by subcluster. The surrounding diagrams show 230 subclusters from 26 main clusters. Top right, UMAP coloured by slice directions; bottom right, UMAP coloured by slice identity as in **c**. **c**, Molecular cell-type maps of the 20 mouse CNS slices coloured by subcluster. Each dot represents one cell. **d**, A zoomed-in view of tissue slice 12 in **c**. Each dot represents a DNA amplicon generated from an RNA molecule, colour-coded by its cell-type identity. Brain region abbreviations are based on the Allen Mouse Brain Reference Atlas^18–20^. alv, alveus; cc, corpus callosum; chpl, choroid plexus; cing, cingulum bundle; CP, caudoputamen; CTX, cerebral cortex; df, dorsal fornix; DG, dentate gyrus; FC, fasciola cinereum; fi, fimbria of hippocampus; HPF, hippocampal formation; int, internal capsule; L2/3, layer 2/3; L4, layer 4; L5, layer 5; L6, layer 6; LH, lateral habenula; MH, medial habenula; mo, molecular layer; po, polymorph layer; RSP, retrosplenial cortex; RT, reticular nucleus of the thalamus; sg, granule cell layer; slm, stratum lacunosum-moleculare; so, stratum oriens; sp, pyramidal layer; sr, stratum radiatum; STR, striatum; TH, thalamus; v3, third ventricle; VL, lateral ventricle. **e**, A zoomed-in view of the habenula region in **d** with cell boundaries outlined (left) and a mesh graph of physically neighbouring cells connected by edges (middle). Symbols for cell types with more than two counts were labelled (right). HABCHO, habenular cholinergic neurons; HABGLU, habenular excitatory neurons; INH, inhibitory neurons; NA, unannotated (see Methods, ‘Main cluster and subcluster cell-type annotation’). **f**, A representative fluorescent image of the region highlighted in **e** from the first cycle of SEDAL. Each dot represents an amplicon.

Here we applied STARmap PLUS^3,4^ to detect 1,022 endogenous genes in 20 CNS tissue slices in situ at a voxel size of 194 × 194 × 345 nm^3^ followed by ClusterMap^12^ cell segmentation. Integrating these data with a published scRNA-seq atlas^1^, we generated molecular cell-type maps based on single-cell gene expression and molecular tissue region maps based on spatial niche gene expression, which enabled a joint nomenclature of brain-wide molecular spatial cell types. Furthermore, we imputed transcriptome-wide, spatially resolved single-cell expression profiles. This work presents a comprehensive molecular spatial atlas of the mouse CNS, comprising more than one million cells with their transcriptome-wide gene expression profiles, spatial coordinates, molecular cell types, molecular tissue regions and joint cell-type nomenclature (Fig. 1a). As an application of the mouse molecular CNS spatial atlas, we developed a highly efficient RNA barcoding system and combined it with STARmap PLUS to chart the tissue and cell-type transduction landscapes of PHP.eB^5,6^, an engineered recombinant adeno-associated virus (rAAV) strain that can penetrate the blood–brain barrier through systemic administration. Together, this work provides experimental and computational frameworks for establishing a molecular spatial atlas across various scales, from individual RNA molecules and single cells to tissue regions.

## Spatial maps of CNS molecular cell types

STARmap PLUS is an image-based in situ RNA sequencing method^3,4^ that uses paired primer and padlock probes (SNAIL probes) to convert target RNA molecules into DNA amplicons with gene-unique codes, which enables highly multiplexed RNA detection in tissue hydrogels by multiple rounds of sequencing by ligation with error rejection (SEDAL) (Fig. 1a).

**Extended Data Fig. 1:**
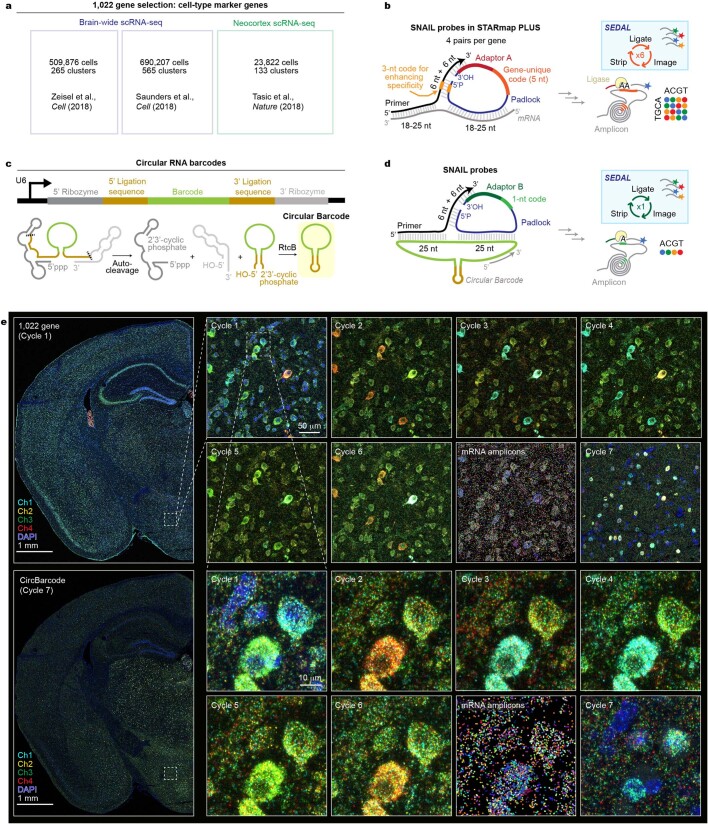
Probe designs and raw fluorescent images of adult mouse CNS STARmap PLUS datasets. **a**, Mouse brain single-cell RNA-seq (scRNA-seq) sources for the STARmap PLUS 1,022 gene-list selection. **b**, SNAIL probes (primer and padlock probes) for 1,022 endogenous genes. The padlock probe contains a 5-nt gene-unique identifier, which is amplified during rolling-circle amplification and read out by six cycles of sequential SEDAL through adaptor sequence *A*. **c**, Schematics showing the construct design and biogenesis of circular RNA barcodes^15^. RtcB, RNA 2’,3’-cyclic phosphate and 5’-OH ligase. **d**, SNAIL probes for circular RNA barcodes. Each barcode is converted to a 1-nt identifier and read out by one additional cycle of SEDAL through adaptor sequence *B*. **e**, Raw fluorescent images of SEDAL of brain slice 12. The left panels show the image stack maximum projection of SEDAL cycles 1 (top) and 7 (bottom), merged into an entire hemisphere slice. The top-right panels show zoomed-in views of SEDAL cycles 1 to 7 and amplicons colored by gene identity from the square highlighted in the left panels. The bottom-right panels show the corresponding zoomed-in views of the square highlighted in the top-right panels.

**Extended Data Fig. 2:**
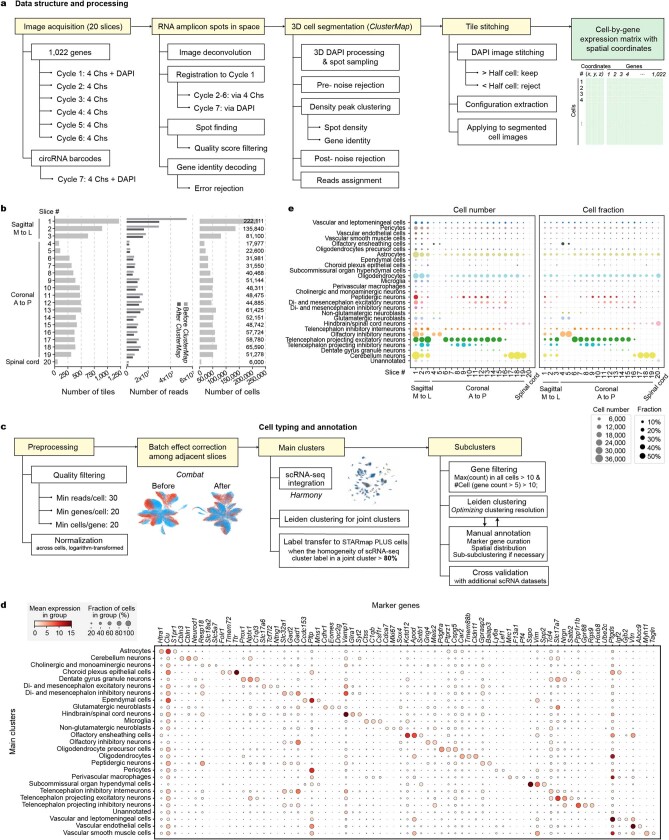
Spatial cell typing workflow and data quality. **a**, Data structure of the study and the workflow from raw images to a cell-by-gene matrix with cell spatial coordinates. Chs, channels. **b**, Summary of the number of tiles (i.e., imaging area), reads and cells in each tissue sample slice. The number of cells is labeled on the figure. **c**, Workflow of cell quality control, batch correction and cell typing. Key parameters and thresholds were labeled. **d**, Dot plots of the top three marker genes for each main cluster. **e**, Main-cluster cell-type composition of each tissue sample slice as in absolute cell number (left) and cell fraction normalized within each tissue slice (right). M, medial; L, lateral; A, anterior; P, posterior. Data are provided in the accompanying Source Data file. Source data

To achieve molecular cell typing, we curated a list of 1,022 genes (Extended Data Fig. 1a and Supplementary Table 1) by compiling reported cell-type marker genes from adult mouse CNS scRNA-seq datasets with minimal post-dissection cell-type selection^1,2,13^. Five-nucleotide codes on the SNAIL probes encoding gene identity were read out by six rounds of SEDAL (Extended Data Fig. 1b and Supplementary Tables 1 and 2). To enable orthogonal detection of adeno-associated virus (AAV) transcripts, we designed highly expressed circular RNA barcodes without homology to the mouse transcriptome^14,15^ (Extended Data Fig. 1c) to be detected in another round of SEDAL (Extended Data Fig. 1d and Supplementary Table 2). We collected STARmap PLUS datasets of 20 10-μm-thick CNS tissue slices from 3 mice, including 16 coronal brain slices, 3 sagittal brain slices and 1 coronal slice from spinal cord lumbar segments (Supplementary Fig. 1a, Supplementary Table 3 and Supplementary Discussion; representative raw fluorescence images in Extended Data Fig. 1e). With an optimized ClusterMap^12^ data processing workflow, we generated a cell-by-gene expression matrix with RNA and cell spatial coordinates (Extended Data Fig. 2a and Methods). In total, the datasets include over 256 million RNA reads and 1.1 million cells (Extended Data Fig. 2b and Supplementary Table 3).

**Extended Data Fig. 3:**
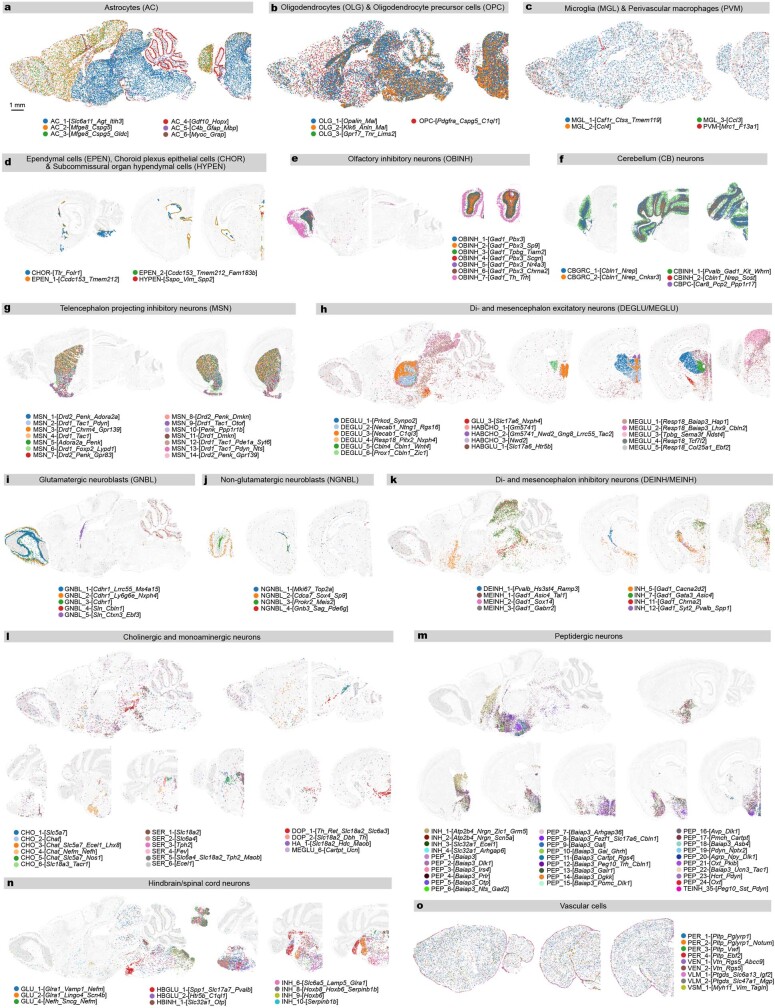
Subclustering of main cell types. **a**-**o**, Subcluster spatial maps on representative sample slices for astrocytes (**a**), oligodendrocytes and oligodendrocyte precursor cells (**b**), microglia and perivascular macrophages (**c**), ependymal cells, choroid plexus epithelial cells and subcommissural organ hypendymal cells (**d**), olfactory inhibitory neurons (**e**), cerebellum neurons (**f**), telencephalon projecting inhibitory neurons (**g**), di- and mesencephalon excitatory neurons (**h**), glutamatergic neuroblasts (**i**), non-glutamatergic neuroblasts (**j**), di- and mesencephalon inhibitory neurons (**k**), cholinergic and monoaminergic neurons (**l**), peptidergic neurons (**m**), hindbrain/spinal cord neurons (**n**), and vascular cells (**o**). Also see Methods and Supplementary Table 4.

**Extended Data Fig. 4:**
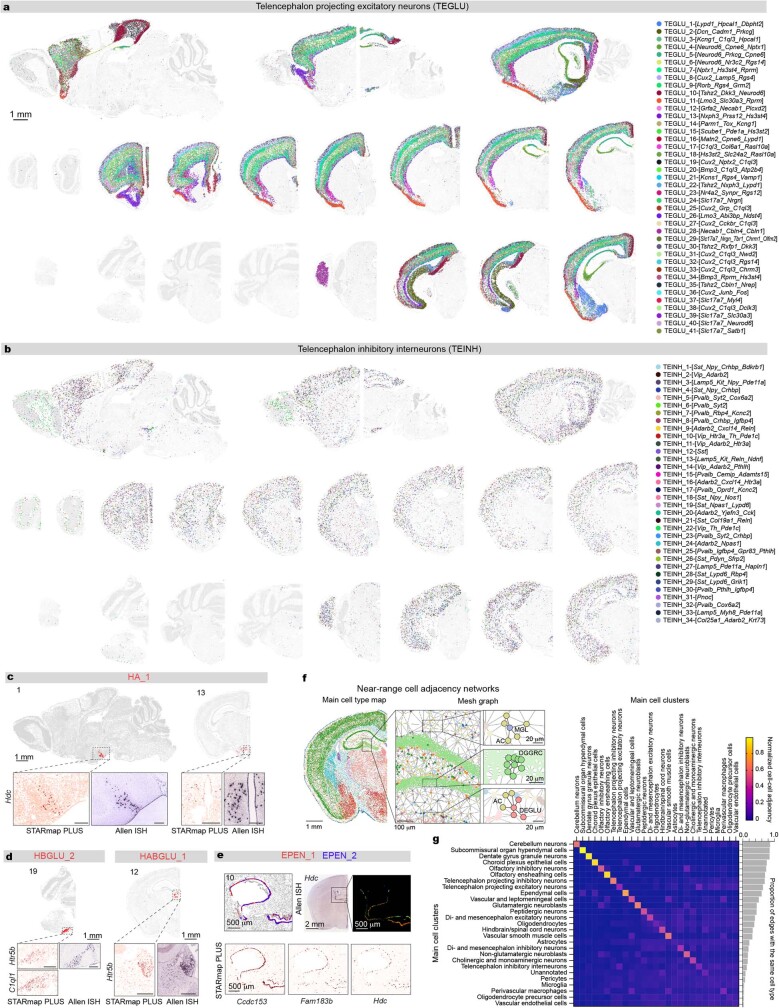
Subclustering of telencephalon projecting excitatory neurons and telencephalon inhibitory interneurons, and spatial maps of representative subcluster cell types. **a**,**b**, Subcluster spatial maps of telencephalon projecting excitatory neurons (TEGLU, **a**) and telencephalon inhibitory interneurons (TEINH, **b**). **c**-**e**, Cell-type spatial maps, zoomed-in spatial expression heatmap of cell-type marker genes measured by STARmap PLUS and corresponding In Situ Hybridization (ISH) images of the marker genes from the Allen Mouse Brain ISH database^23^, for subcluster cell types HA_1 (**c**), HBGLU_2 and HABGLU_1 (**d**) and EPEN_1 and EPEN_2 (**e**). Each dot represents a cell colour-coded by its subcluster cell-type symbol. Scale bars, 250 μm if not indicated. **f**, Mesh graph of cells shown on the STARmap PLUS molecular cell type map. Each cell is represented by a spot in the colour of its corresponding main cell type. Physically neighboring cells are connected via edges. Zoomed-in views of the top, middle, and bottom squares in the middle are shown on the right. **g**, First-tier cell-cell adjacency quantified by the normalized number of edges between individual pairs of main cell types (left). For each main cell type, the proportion of edges formed with cells of the same main type over the total number of edges with adjacent cells is shown in the bar plot (right). HA, histaminergic neurons; HBGLU, hindbrain excitatory neurons; HABGLU, habenular excitatory neurons; EPEN, ependymal cells; AC, astrocytes; MGL, microglia; DGGRC, dentate gyrus granule cells; DEGLU, diencephalon excitatory neurons. Also see Methods and Supplementary Table 4. Data are provided in the accompanying Source Data file. Source data

After batch correction, we pooled cells from all the tissue slices and performed cell typing by hierarchically clustering single-cell expression profiles (Extended Data Fig. 2c and Methods). To annotate cell types and align them with published cell-type nomenclature^1,2,13^, we integrated our data with an existing mouse CNS scRNA-seq atlas^1^ using Harmony^16^. Leiden clustering followed by nearest-neighbour label transfer identified 26 main cell types, including 13 neuronal, 7 glial, 2 immune and 4 vascular cell clusters, all of which exhibit canonical marker genes and expected spatial distribution across the 20 tissue slices (Fig. 1b and Extended Data Figs. 2d,e, 3 and 4). Further Leiden clustering within each main cluster resulted in 230 subclusters, including 190 neuronal, 2 neural crest-like glial, 13 CNS glial, 4 immune and 9 vascular cell clusters (Fig. 1b and Supplementary Figs. 1b–d, 2 and 3). We annotated each subcluster with symbols, cell counts, marker genes and spatial distributions, and indicated whether they represent cell types or states (Supplementary Table 4). Notably, the subcluster size in our data spans approximately three orders of magnitude, ranging from abundant cell types such as oligodendrocytes (OLGs) (OLG_1; 70,866 cells, 6.5% of total cells; Extended Data Fig. 3b and Supplementary Fig. 2b) to rare cell types such as *Hdc*^*+*^ histaminergic neurons^17^ in the posterior hypothalamus (HA_1; 111 cells, 0.01% of total cells; Extended Data Figs. 3l and 4c and Supplementary Fig. 2i).

We then plotted molecularly defined, single-cell resolved cell-type maps across the adult mouse CNS (Fig. 1c and Extended Data Figs. 3 and 4a,b). Notably, the maps clearly delineate brain structures, including the cerebral cortex (41 telencephalon projecting excitatory neurons (TEGLU) and 34 telencephalon inhibitory interneurons (TEINH neuron types)), olfactory bulb (7 olfactory inhibitory neurons (OBINH neuron types) and olfactory ensheathing cells (OEC)), striatum (14 telencephalon projecting inhibitory neurons (or medium spiny neurons; MSN)), cerebellum (5 cerebellum neuron types and astrocyte type AC_4), and brainstem (29 peptidergic, 16 cholinergic and monoaminergic, 16 di- and mesencephalon excitatory, 8 di- and mesencephalon inhibitory and 10 hindbrain/spinal cord neuron types), fully recapitulating the anatomical regions in the adult mouse CNS^18–20^ (Fig. 1c). Zooming in of these maps also reveals cell-type-specific patterns in fine tissue regions, such as the medial and lateral habenula, alveus, fimbria and ependyma (Fig. 1d), with individual cells (Fig. 1e) and RNA molecules (Fig. 1f) fully resolved in space.

Compared with previous scRNA-seq results^1,2^, the molecular resolution, single-cell mapping across a large number of cells enables more precise annotation of molecular cell types by their spatial distributions. For instance, in addition to the previously reported *Htr5b*^+^ neurons^1^ in the inferior olivary complex of the hindbrain (HBGLU_2, *Slc17a6*^+^*C1ql1*^+^, 204 cells), we identified another *Htr5b*^+^ cluster located in the habenula (HABGLU_1, *Slc17a6*^+^*C1ql1*^−^, 318 cells) (Extended Data Fig. 4d and Supplementary Fig. 2h). We also observed that ependymal cells (EPEN) contain two subclusters (EPEN_1, *Ccdc153*^+^; EPEN_2, *Ccdc153*^+^*Fam183b*^+^) with differential distributions across the medial-lateral axis (Extended Data Fig. 4e and Supplementary Fig. 2d). Moreover, our single-cell-resolved molecular cell-type maps enabled us to examine cell–cell adjacency across the entire brain (Fig. 1e and Extended Data Fig. 4f), revealing that neuronal cell types tend to form near-range networks with the same main cell type, whereas glial and immune cell types are more sparsely distributed among other cell types (Extended Data Fig. 4g and Supplementary Table 4). In brief, our molecular resolution, large-scale in situ sequencing data provide substantial potential for annotating molecular cell types and characterizing cellular neighbourhoods in space.

## Molecularly defined CNS tissue regions

**Fig. 2:**
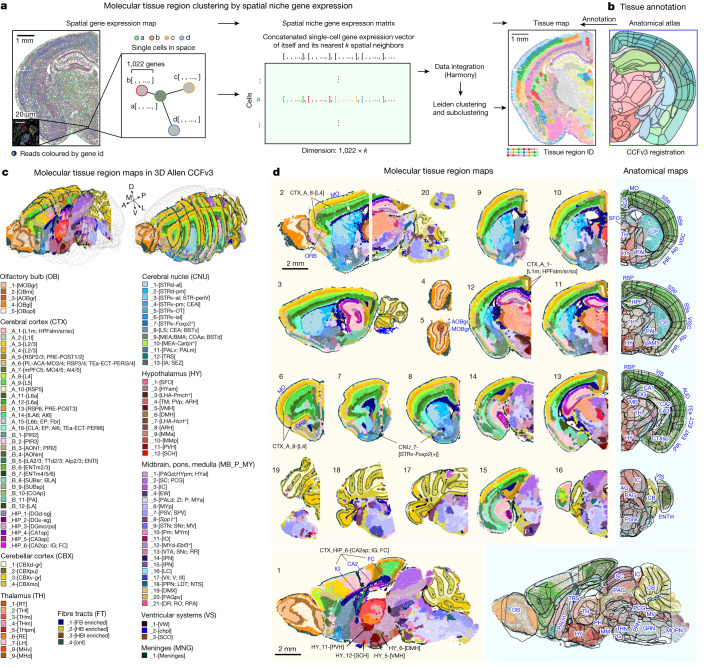
Molecular tissue regions across the adult mouse CNS. **a**, Schematics of the workflow of clustering molecular tissue regions by single-cell resolved spatial niche gene expression. A spatial niche gene expression vector of each cell was formed by concatenating its single-cell gene expression vector and those of the *k*NNs in physical space. The vectors of all cells were stacked into a spatial niche gene expression matrix and Leiden-clustered into molecular tissue regions. **b**, Allen Mouse Brain Common Coordinate Framework^20^ (CCFv3, 10 μm resolution) registration to facilitate molecular tissue region annotation. **c**,**d**, Molecular tissue region maps registered into the visualizations in 3D (**c**; 16 coronal and 3 sagittal brain slices combined) and 2D (**d**; individual slices). Representative registrations are shown to compare corresponding molecular tissue regions with anatomical tissue regions (anatomical outlines on top of molecular cell-type maps) on the same slice (**d**, right). Each dot represents a cell. Anatomical region definitions are labelled in blue. Tissue region abbreviations are based on the Allen Mouse Brain Reference Atlas^18–20^ (Supplementary Notes). ACA, anterior cingulate area; Alp, posterior agranular insular area; AOBgr, accessory olfactory bulb, granule layer; AQ, cerebral aqueduct; AUD, auditory areas; CTXsp, cortical subplate; ECT, ectorhinal area; ENT, entorhinal area; ENTm, entorhinal area, medial part; GRN, gigantocellular reticular nucleus; HY, hypothalamus; IC, inferior colliculus; IG, indusium griseum; MB, midbrain; MDRN, medullary reticular nucleus; MM, medial mammillary nucleus; MO, somatomotor areas; MOBgr, main olfactory bulb, granular layer; MV, medial vestibular nucleus; ORB, orbital areas; PAG, periaqueductal gray; PAL, pallidum; PALm, pallidum, medial region; PG, pontine gray; PH, posterior hypothalamic nucleus; PIR, piriform area; PRN, pontine reticular nucleus; RSP, retrosplenial area; sAMY, striatum-like amygdalar nuclei; SC, superior colliculus; SFO, subfornical organ; SSp, primary somatosensory area; SSs, supplemental somatosensory area; TEa, temporal association areas; TRN, tegmental reticular nucleus; TRS, triangular nucleus of septum; VIS, visual areas; VISC, visceral area.

**Extended Data Fig. 5:**
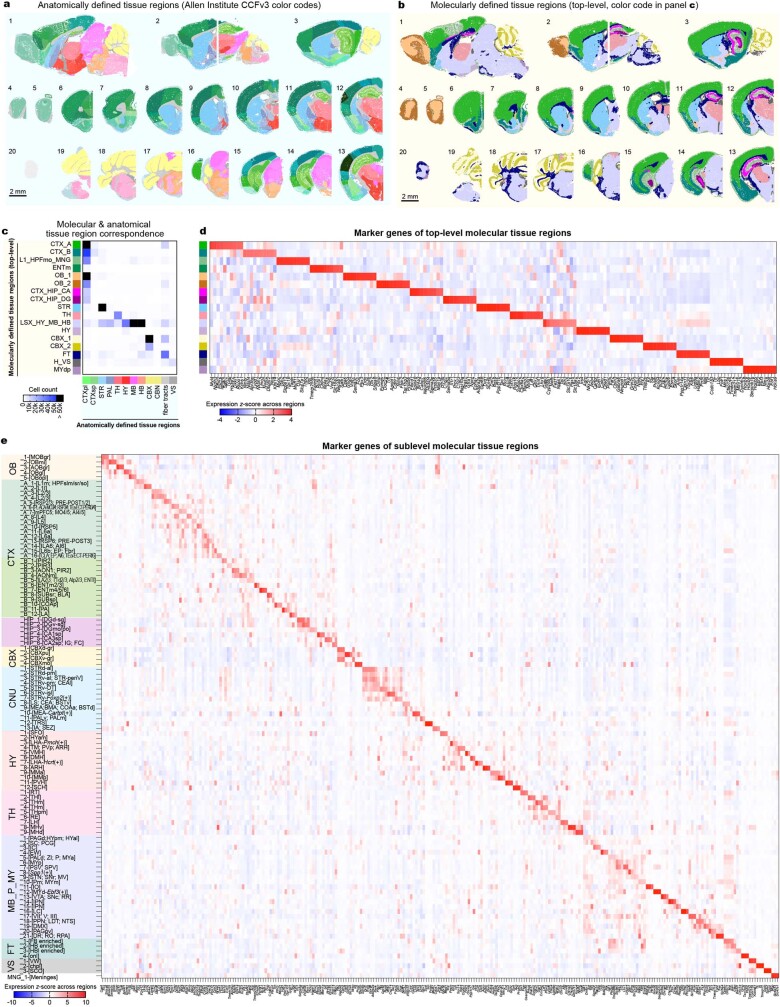
Brain anatomy registration (Allen CCFv3) and marker genes of molecular tissue regions. **a**,**b**, Spatial plots of 20 sample slices coloured by CCF anatomical labels according to the Allen Institute 3D Mouse Brain Atlas^20^ (**a**) and top-level molecularly defined tissue regions (**b**). Each dot represents a cell. **c**, Heatmap showing the correspondence between main anatomical regions and top-level molecularly defined tissue regions. **d**,**e**, Marker gene heatmaps for top-level molecular tissue regions (top ten markers per region, ranked by *z*-scores of mean expression across regions, **d**) and sublevel molecular tissue regions (top three markers per region, ranked by *z*-scores of mean expression across regions, **e**). Tissue region abbreviations: OB, olfactory bulb; CTX, cerebral cortex; CBX, cerebellar cortex; CNU, cerebral nuclei; TH, thalamus; HY, hypothalamus; MB_P_MY, midbrain, pons, and medulla; FT, fibre tracts; VS, ventricular systems; H, habenula; MYdp, medulla, dorsoposterior part; HPFmo, non-pyramidal area of hippocampal formation; MNG, meninges; ENTm, entorhinal area, medial part; HIP, hippocampal region; DG, dentate gyrus; STR, striatum; CTXpl, cortical plate; LSX, lateral septal complex; PAL, pallidum; HB, hindbrain; CBN, cerebellar nuclei. Data are provided in the accompanying Source Data file. Source data

Next, we built molecularly defined tissue region maps directly from spatial niche gene expression profiles. Such data-driven identification of tissue regions provides systematic and unbiased molecular definitions of CNS tissue domains^8^. In brief, for a given tissue slice, a spatial niche gene expression vector of each cell was formed by concatenating its own single-cell gene expression vector and those of its *k*-nearest neighbours (*k*NNs) in the physical space. The resulting spatial niche gene expression matrices for each slice were integrated and subjected to Leiden clustering (Fig. 2a and Methods) to identify major brain tissue regions (17 top-level clusters) and then subclusters within each major region (106 sublevel clusters). To compare and annotate the molecularly defined tissue regions with anatomically defined tissue regions, we registered sample slices into the established Allen Mouse Brain Common Coordinate Framework^20–22^ (CCFv3) (Fig. 2b,c) and labelled individual cells in our datasets with CCF anatomical definitions (Extended Data Fig. 5a and Methods).

Overall, the molecularly defined tissue regions aligned well with the anatomically defined regions (Fig. 2d and Extended Data Fig. 5a–c) and were annotated accordingly. First, the identified marker genes in each top-level molecular tissue region were consistent with region markers reported in the Allen In Situ Hybridization (ISH) database^23^ (Extended Data Fig. 5d and Supplementary Table 5), such as the molecular dentate gyrus marker *C1ql2*, the molecular striatal marker *Ppp1r1b* and the molecular thalamic marker *Tcf7l2*. Next, the 106 sublevel clusters comprise 5 molecular olfactory bulb regions (OB_1–5), 34 molecular cerebral cortex regions (CTX_A_1 – 16, CTX_B_1 – 12 and CTX_HIP_1 – 6), 13 molecular cerebral nuclei regions (CNU_1 – 13), 4 molecular cerebellar cortex regions (CBX_1 – 4), 9 molecular thalamic regions (TH_1 – 9), 12 molecular hypothalamic regions (HY_1 – 12), 21 molecular tissue regions in the midbrain, pons and medulla (MB_P_MY_1 – 21), 4 molecular fibre-tract regions (FT_1 – 4), 3 molecular ventricular system regions (VS_1 – 3) and the molecular meninges (MNG_1). We subsequently annotated individual sublevel molecular tissue regions with symbols describing fine anatomical definitions, preferential distribution along body axes (anterior versus posterior, medial versus lateral and dorsal versus ventral) or marker genes (Extended Data Fig. 5e and Supplementary Table 5), following the anatomical nomenclature in the Allen Institute Adult Mouse Atlas^18–20^ (Fig. 2d). For example, OB_1 corresponds to the granule layer of the main olfactory bulb and is thus named OB_1-[MOBgr].

**Extended Data Fig. 6:**
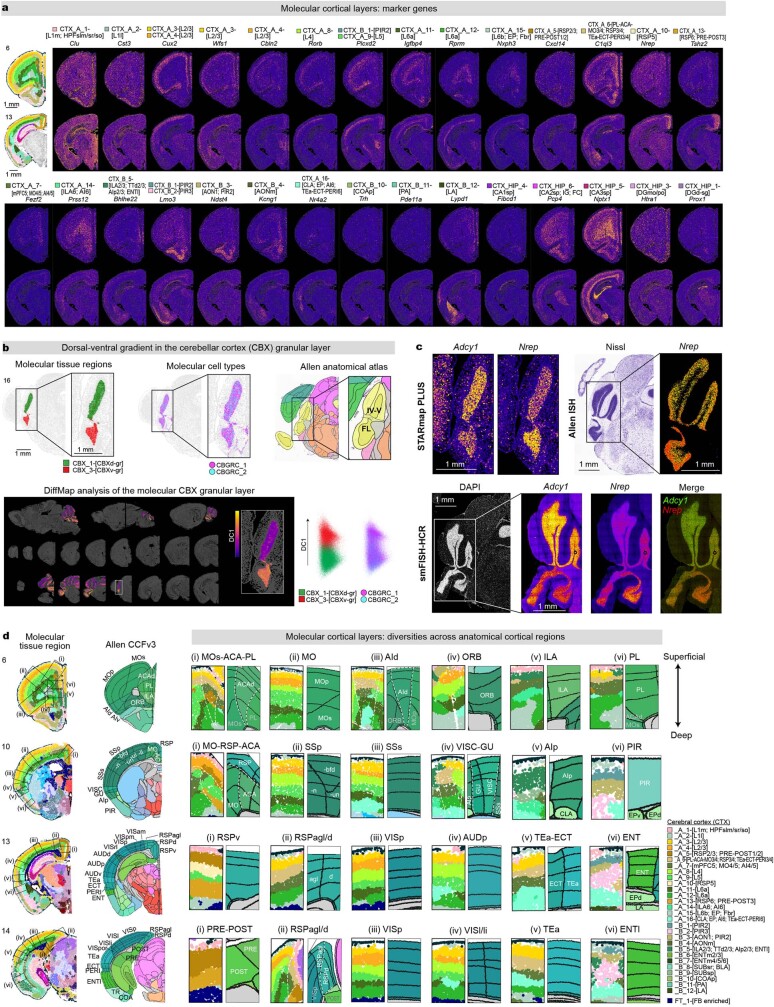
Molecular diversity within the cerebral cortex and the cerebellar cortex granular layer. **a**, Spatial expression heatmap of representative marker genes for molecular cerebral cortical regions. **b**, Molecular tissue regions, molecular cell types and anatomical definition maps (from the Allen Mouse Brain Atlas^18,19^) at the cerebellar cortex granule layer (top), spatial maps of molecular cerebellar cortex granule layer coloured by the value of the first eigenvector of the diffusion map (DC1) (bottom left), and DC embeddings of spatial niche gene expression coloured by molecular tissue region identities (bottom middle) or molecular cell type identities (bottom right). IV-V, culmen lobules IV-V; FL, flocculus. **c**, STARmap PLUS, Allen ISH^23^ and smFISH-HCR images of *Adcy1* and *Nrep* that are enriched in the dorsal and ventral parts of the cerebellar cortex granular layer (CBX_1-[CBXd_gr] versus CBX_3-[CBXv_gr]), respectively. smFISH-HCR images are representative of two experiments. **d**, Comparison of the molecular and anatomical tissue layer composition in various cortical regions covering the anterior-posterior, lateral-medial, and dorsal-ventral axes. Anatomical maps were shown as the registered tissue slices in CCFv3^20^. Anatomical tissue region abbreviations: MO, somatomotor areas; MOs, secondary motor area; MOp, primary motor area; ACA, anterior cingulate area; ACAd, anterior cingulate area, dorsal part; PL, prelimbic area; AId, agranular insular area, dorsal part; AIp, agranular insular area, posterior part; ORB, orbital area; ILA, infralimbic area; RSP, retrosplenial area; RSPv, retrosplenial area, ventral part; RSPagl, retrosplenial area, lateral agranular part; RSPd, retrosplenial area, dorsal part; SSp, primary somatosensory area; SSs, supplemental somatosensory area; VISC, visceral area; GU, gustatory areas; PIR, piriform area; VISam, anteromedial visual area; VISpm, posteromedial visual area; VISp, primary visual area; VISrl, rostrolateral visual area; VISl, lateral visual area; VISli, laterointermediate area; VISpor, postrhinal area; AUDd, dorsal auditory area; AUDp, primary auditory area; AUDv, ventral auditory area; TEa, temporal association areas; ECT, ectorhinal area; PERI, perirhinal area; ENT, entorhinal area; ENTl, entorhinal area, lateral part; TR, postpiriform transition area; COA, cortical amygdalar area; PRE, presubiculum; POST, postsubiculum. Molecular tissue region abbreviations, see Supplementary Notes and Supplementary Table 5.

We carefully examined our molecular tissue annotation and marker genes by cross-referencing published studies and validating with single-molecule fluorescence in situ hybridization with hybridization chain reaction amplification^24^ (smFISH–HCR). First, the molecular cerebral cortical regions resemble the laminar organization of anatomical cortical layers^8,11^ and recapitulate layer-specific markers (for example, *Cux2* in CTX_A_3-[L2/3] and CTX_A_4-[L2/3], *Rorb* in CTX_A_8-[L4], *Plcxd2* in CTX_A_9-[L5] and *Rprm* in CTX_A_12-[L6a]) (Fig. 2d and Extended Data Fig. 6a). Second, in the hippocampal region, we observed expected markers for the pyramidal layers in individual Ammon’s horn fields, including *Fibcd1* in CTX_HIP_4-[CA1sp], *Pcp4* in CTX_HIP_6-[CA2sp; IG; FC] and *Nptx1* in CTX_HIP_5-[CA3sp] (Fig. 2d, slices 1–3 and 11–15, and Extended Data Fig. 6a). Third, both molecular olfactory bulb regions (OB_1 – 5) and molecular cerebellar cortical regions (CBX_1 – 4) form delicate layered structures corresponding to anatomically defined layers (Fig. 2d, olfactory bulb: slices 1, 2, 4 and 5; cerebellum: slices 1–3 and 16–19). Notably, molecular tissue regions further reveal gene expression differences between the granule layers of the main and accessory olfactory bulb (OB_1-[MOBgr] versus OB_3-[AOBgr], marked by *Inpp5j* and *Trhr*, respectively; Fig. 2d, slice 5) and between the dorsal and ventral CBX granular layer^25^ (CBX_1-[CBXd-gr] versus CBX_3-[CBXv-gr], marked by *Adcy1* and *Nrep*, respectively; Fig. 2d, slices 1–3 and 16–19 and Extended Data Fig. 6b,c). Fourth, multiple subdivisions of the molecular regions in thalamus and hypothalamus appear as spatially segregated nuclei, corresponding to anatomically defined structures distributed along body axes (Fig. 2d, slices 1 and 11–13), such as the *Six3*^+^ reticular nuclei of thalamus (TH_1-[RT]), the *Spon1*^+^ nucleus of reuniens of thalamus (TH_6-[RE]), the *Chrna3*^+^ ventral medial habenula (TH_8-[MHv]), the *Fezf1*^+^ ventromedial hypothalamic nucleus (HY_5-[VMH]), the *Oxt*^+^ paraventricular hypothalamic nucleus (HY_11-[PVH]), the *Ppp1r17*^+^ dorsomedial nucleus of the hypothalamus (HY_6-[DMH]), the *Agrp*^+^ arcuate hypothalamic nucleus (HY_8-[ARH]) and the *Prokr2*^+^ hypothalamic suprachiasmatic nucleus (HY_12-[SCH]) (Fig. 2d and Extended Data Fig. 5e). Finally, in the midbrain and hindbrain, we were able to capture gene signatures in fine structures of brain nuclei, such as *Cartpt* in the Edinger–Westphal nucleus (MB_P_MY_4-[EW]), *Dbh* in the locus coeruleus (MB_P_MY_16-[LC]) and *Chrna2* in the apical interpeduncular nucleus (MB_P_MY_14-[IPN]) (Fig. 2d and Extended Data Fig. 5e).

However, molecularly defined tissue regions are not necessarily the same as anatomically defined tissue regions. Molecular tissue regions illustrate molecular spatial heterogeneity that lacks obvious anatomical borders—for example, the molecular cortical layer maps reveal the similarity and differences in molecular compositions among various cortical regions across the medial–lateral and anterior–posterior axes^26^ (Fig. 2d and Extended Data Fig. 6d). Specifically, previous studies have indicated a putative cortical layer 4 (L4) in the motor cortex^11,27^, whose existence was supported by our molecular tissue regions (CTX_A_8-[L4], marked by *Rorb* and *Rspo1*). We showed further that L4 also exists in the orbital area (ORB) (Fig. 2d, slices 2 and 6). Additionally, previous studies^2,10^ have identified atypical *Foxp2*^+^ D1 MSN cell types in the striatum. Our data further illustrate a unique molecular tissue region (CNU_7-[STRv_*Foxp2*^+^]) that contains *Foxp2*^+^ D1 MSNs and forms patch-like structures at the boundary of the ventral striatum (Fig. 2d, slices 2–3 and 7–11). Conversely, molecular tissue regions reveal spatial gene expression similarities among multiple anatomically defined regions. For example, our data suggest similar spatial expression profiles in the medial cortical layer 1 and hippocampal molecular layers (CTX_A_1-[L1m; HPFslm/sr/so]; Fig. 2d, slice 12), probably related to the parallel correlation between the isocortex and allocortex^26^. As another example, indusium griseum (IG) and fasciola cinereum (FC) are two small subregions in the hippocampal region. Given their similarity in cytoarchitecture to the dentate gyrus (DG), whether they constitute unique subregions or belong to dentate gyrus is still under debate^28^. Our molecular tissue regions suggest that, with respect to spatial gene expression, both indusium griseum and fasciola cinereum exhibit high resemblance with CA2 (CTX_HIP_6-[CA2sp; IG; FC], high in *Rgs14* and *Cabp7*; Fig. 2d, slices 1, 8, 11 and 12), supporting the observed similarity among CA2, indusium griseum and fasciola cinereum in the expression of key proteins^26,28^, but precluding that they are remnants of the DG^29,30^.

Collectively, we report a resource of molecular tissue regions across the mouse CNS registered with brain anatomy and annotated with region-specific marker genes (Supplementary Table 5). The general match of molecular and anatomical tissue regions confirms the molecular basis of mouse brain anatomy. More importantly, this unbiased identification of molecular tissue regions enables the discovery of new tissue architectures that complement the established brain anatomy, as further illustrated in the subsequent joint analysis of molecular cell types and tissue regions.

## Joint molecular cell types and regions

**Fig. 3:**
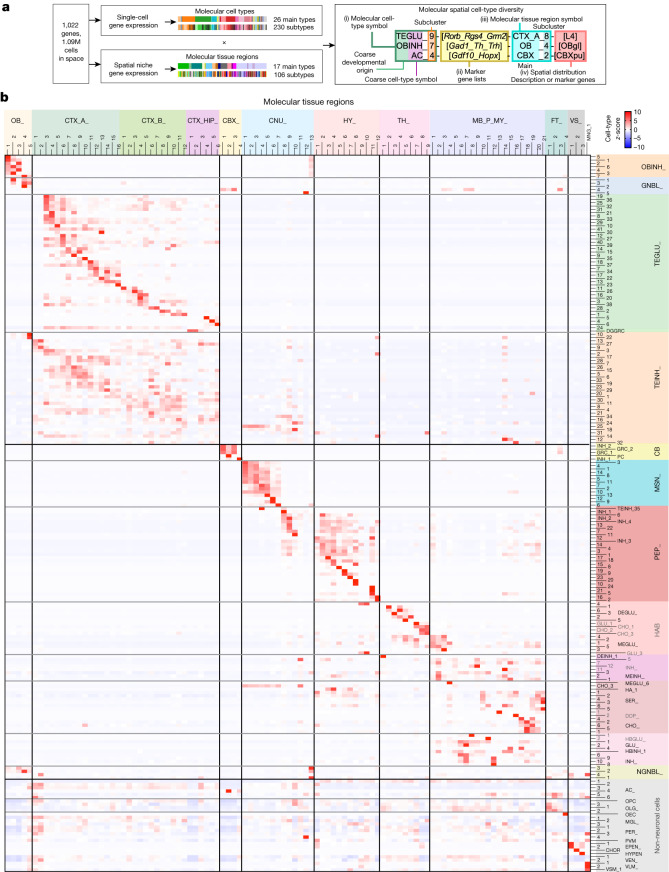
Joint nomenclature of cell clusters through the combination of molecular cell types and molecular tissue regions. **a**, Schematic illustrating the workflow that combines molecular cell types and molecular tissue regions to jointly define cell-type nomenclatures. **b**, Heat map showing the distribution of molecular cell types across molecular tissue regions. The cell-type percentage composition is calculated for each molecular tissue region. Then for each cell type, the *z*-scores of its percentages across regions are plotted. Subtypes of the same main cell type are grouped together. CBX, cerebellar cortex; CHO, cholinergic neurons; CNU, cerebral nuclei; DOP, dopaminergic neurons; FT, fibre tracts; HA, histaminergic neurons; HAB, habenular cells; HBGLU, hindbrain excitatory neurons; HBINH, hindbrain inhibitory neurons; MB_P_MY, midbrain, pons and medulla; OB, olfactory bulb; SER, serotonergic neurons; VS, ventricular systems. See also Fig. 1b. Molecular tissue region abbreviations are provided in Supplementary Notes. Data are provided in the accompanying Source Data file. Source data

**Extended Data Fig. 7:**
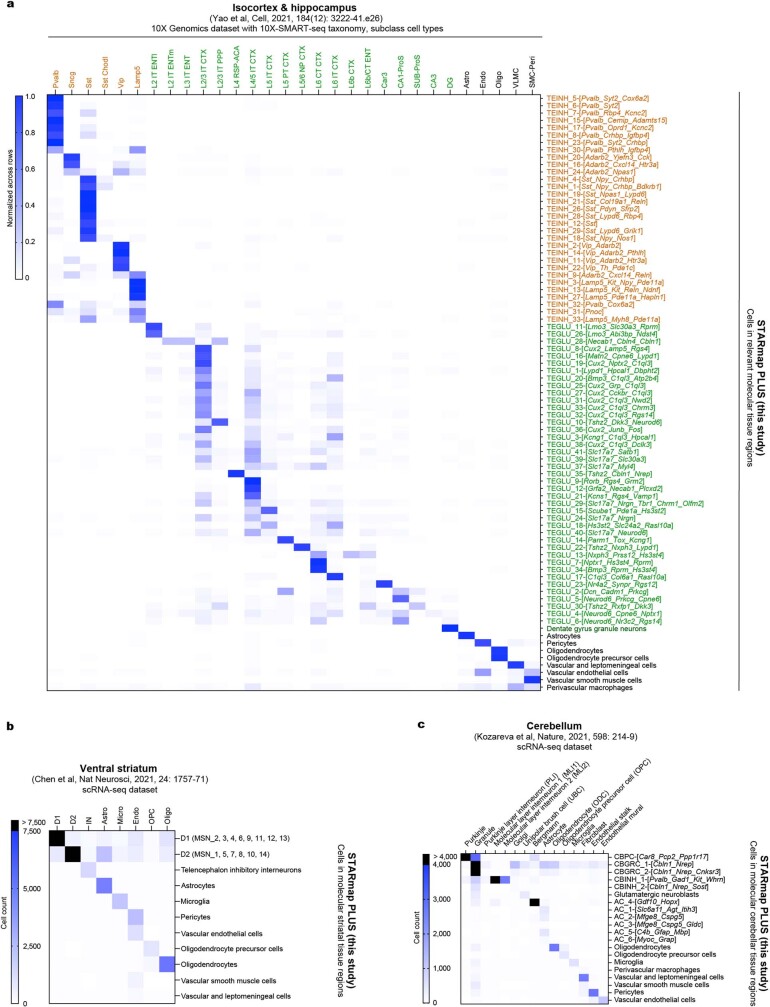
Cross-reference correspondence of STARmap PLUS main and subcluster cell types. Cell-type correspondence to cell types annotated in scRNA-seq datasets of adult mouse brain subregions including datasets on isocortex and hippocampus from the Allen Institute^26^ (**a**), ventral striatum (nucleus accumbens^10^, **b**) and cerebellum^25^ (**c**). Cell type abbreviations: IT, intratelencephalic; PT, pyramidal tract; NP, near-projecting. Data are provided in the accompanying Source Data file. Source data

Next, we created a comprehensive molecular spatial cell-type nomenclature by combining information on molecular cell type, subtype, marker genes and molecular tissue region distribution for each cell (Fig. 3a), resulting in 1,997 molecular spatial cell types (Supplementary Table 6). This joint definition enabled us to further validate our annotated molecular cell types by cross-referencing scRNA-seq studies on subregions of the adult mouse brain. Indeed, we observed good correspondence between our cell clusters and neuronal and glial cell types in regional scRNA-seq results for the isocortex and hippocampus^26^, ventral striatum^10^ and cerebellum^25^ (Extended Data Fig. 7).

**Extended Data Fig. 8:**
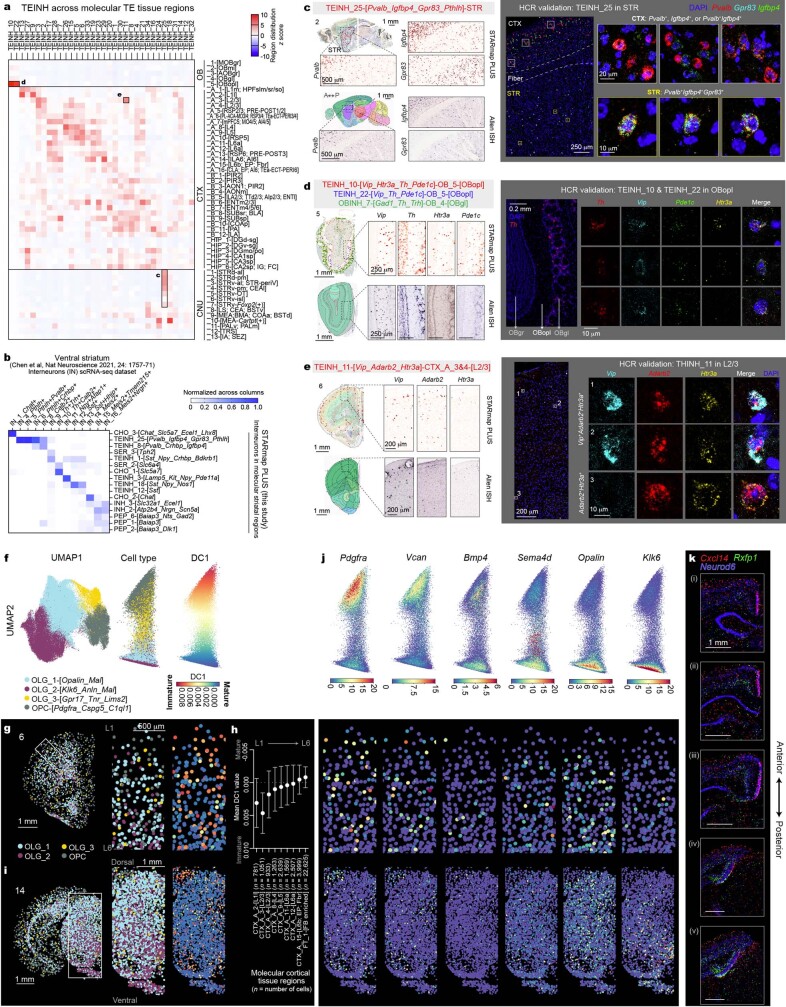
Joint analysis and validation of molecular cell clusters in molecular tissue regions. **a**, Heatmap showing the distribution of telencephalon inhibitory interneuron (TEINH) cell types across molecular telencephalon (TE) tissue regions. **b**, Correspondence of interneuron subtypes within the molecular striatal tissue regions to interneuron (IN) cell types annotated in the scRNA-seq dataset of adult mouse ventral striatum (nucleus accumbens)^10^. **c**-**e**, Cell type maps overlaid on molecular tissue regions, spatial expression heatmap of cell-type marker genes measured by STARmap PLUS, corresponding ISH images of the marker genes from the Allen Mouse Brain ISH database^23^ and independent smFISH-HCR validation of the distribution of the positive cells for TEINH_25 in the striatum (**c**), TEINH_10 and TEINH_22 in the olfactory bulb outer plexiform layer (OBopl, **d**) and TEINH_11 in cerebral cortical layer 2/3 (**e**). smFISH-HCR images are representative of two experiments (**c**-**e**). The ISH data were obtained from Allen Mouse Brain Atlas. **f**, UMAP embedding of OPC and OLG (left) and DC embedding^69^ coloured by molecular cell types (middle) and DC1 value (right). **g**,**i**, Spatial distribution of DC1 values of the OPC-OLG lineage and OPC-OLG molecular cell cluster identities in the cerebral cortical layers (**g**) and midbrain-pons dorsal-ventral axis (**i**). **h**, DC1 values of the OPC-OLG lineage across the molecular cortical layers. Data shown as mean ± s.t.d. **j**, DC embedding (top) and spatial maps (bottom) coloured by marker gene expression levels indicating oligodendrocyte differentiation and maturation states. Only OPC and OLG cells are plotted (**g**,**i**,**j**). **k**, STARmap PLUS expression heatmap of *Cxcl14*, *Rxfp1*, and *Neurod6* in representative coronal slices along the anterior-posterior axis. Data are provided in the accompanying Source Data file. Source data

Using these spatially resolved cell-type labels, we systematically examined the spatial distribution of cell types across brain regions (Fig. 3b and Supplementary Table 5). In the cerebral cortex, we observed a strong layer-specific distribution of TEGLU neuron types (Fig. 3b) as previously reported^11,26^. In addition, our data showed that modest layer preference of TEINH neuron types exists across cortical areas (Fig. 3b) beyond previously reported primary visual cortex^3^ and primary motor cortex^11^. Our data also revealed new region-specific TEINH subtypes (Extended Data Fig. 8a), which we further verified through smFISH–HCR^24^ as follows. We identified and experimentally validated (1) a striatum-specific interneuron subtype, TEINH_25-[*Pvalb_Igfbp4_Gpr83_Pthlh*], which has been indicated in a previous single-cell RNA-seq study comparing cortical and striatal interneurons^31^ and a recent striatum scRNA-seq dataset^10^ (Extended Data Fig. 8b,c); (2) two *Th*^+^*Vip*^+^ interneuron subtypes, TEINH_10-[*Vip_Htr3a_Th_Pde1c*] and TEINH_22-[*Vip_Th_Pde1c*], which are restrictively located in the outer plexiform layer of the olfactory bulb (OB_5-[OBopl]) (Extended Data Fig. 8a,d) and distinct from the previously identified olfactory glomerular layer *Th*^+^*Vip*^−^ interneurons^32^ (OBINH_7-[*Gad1_Th_Trh*]); and (3) a L2/3 enriched subtype, TEINH_11-[*Vip_Adarb2_Htr3a*] (Extended Data Fig. 8a,e). Furthermore, many neuronal cell types outside the cerebral cortex also exhibit defined spatial patterns (Fig. 3b and Extended Data Fig. 3). We observed differential distributions of OBINH cell types across the layers in the olfactory bulb and GBNL cell types enriched at the mitral (OBmi) and glomerular (OBgl) layers. In the brainstem, we identified molecular tissue regions enriched with distinct neuronal types, such as DEINH_1-[*Pvalb*_*Hs3st4_Ramp3*] in TH_1-[RT] and DEGLU_3-[*Necab1_C1ql3*] in the dorsal–medial thalamus TH_3-[THm] (Fig. 3b and Extended Data Fig. 3h,k).

Although many glial cell types did not show strong tissue region-specific distribution (Fig. 3b) as expected^11,26^, we observed a few exceptions. First, our results confirmed previous reports of region-specific enrichment of astrocyte subtypes^1^, including those in the telencephalon (AC_2,3), non-telencephalon (AC_1), cerebellar Purkinje cell layer (AC_4), fibre tracts (AC_5), and meninges (AC_6) (Fig. 3b and Extended Data Fig. 3a). Second, we examined the region-specific distribution of the OLG lineage, including oligodendrocyte precursor cell (OPC) and OLG_1–3. The results showed that (1) in the cerebral cortex, OPC-OLG cells in deeper layers tend to be more mature; and (2) the hindbrain contains a higher percentage of OLGs at more mature stages than the forebrain and midbrain (Extended Data Fig. 8f–i), which aligns with the recent finding that the ratio of OLGs to OPCs is higher in the human brainstem than in other regions^33^.

**Fig. 4:**
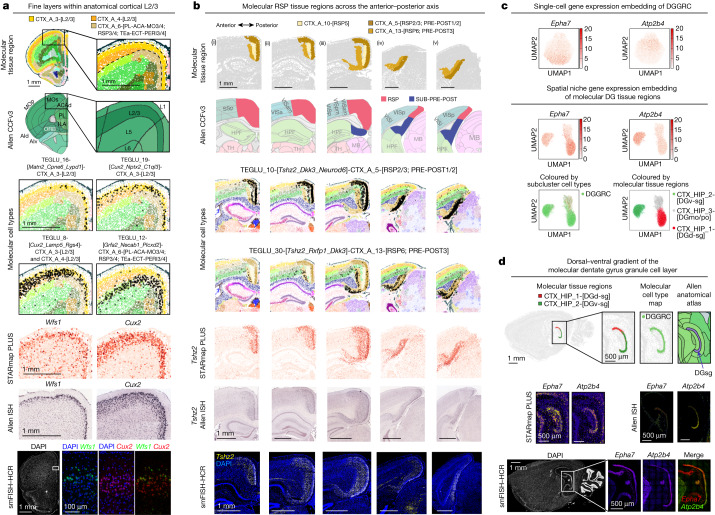
Joint analysis and validation of molecular cell types in molecular tissue regions. **a**,**b**, From top to bottom: molecular tissue region maps, anatomical tissue maps registered to Allen CCFv3 (ref. ^20^), marker cell-type distribution maps (cells within the specified region marked in dots, otherwise in ‘×’), marker gene STARmap PLUS measurements, marker gene Allen Mouse Brain ISH expression^23^ and smFISH–HCR validation of molecular cortical superficial laminar structure (CTX_A_3-[L2/3]) within the anatomical cortical L2/3 (**a**) and anterior–posterior (i–v) distribution of molecular RSP tissue regions (**b**). Cortical areas adjacent to RSP are labelled in the anatomical tissue maps. ACAd, anterior cingulate area, dorsal part; ILA, infralimbic area; MOp, primary motor area; MOs, secondary motor area; PL, prelimbic area; POST, postsubiculum; PRE, presubiculum; SUB, subiculum. **c**, *Epha7* and *Atp2b4* expression plotted in the single-cell gene expression UMAP of DGGRCs (top) and the spatial niche gene expression UMAP of molecular DG regions (middle), and spatial niche gene expression UMAP coloured by molecular cell types and molecular DG sublevel tissue regions (bottom). DGd-sg, dentate gyrus granule cell layer, dorsal part; DGv-sg, dentate gyrus granule cell layer, ventral part. **d**, Molecular tissue region map, molecular cell-type map and anatomical region map of dentate gyrus granule cell layer (DGsg) (top), STARmap PLUS measurements and Allen ISH expression (middle)^23^, and smFISH–HCR validation (bottom) of *Epha7* and *Atp2b4*. smFISH–HCR images are representative of two (**a**,**d**) and three (**b**) experiments. The ISH data were obtained from the Allen Mouse Brain Atlas^23^.

More importantly, we found tissue structures that differ from brain anatomy described in CCFv3, along with associated cell types and gene markers. First, molecular tissue regions illustrate spatial gene expression patterns that are not captured by anatomical structures, such as a fine lamina (CTX_A_3-[L2/3]) in the superficial layer of anatomical cerebral cortical L2/3 (Fig. 4a) marked by high expression of *Wfs1* and enriched with molecular cell types TEGLU_16-[*Matn2_Cpne6_Lypd1*] and TEGLU_19-[*Cux2_Nptx2_C1ql3*]. By contrast, the canonical L2/3 marker *Cux2* (ref. ^11^) occupies molecular tissue regions CTX_A_3-[L2/3] and CTX_A_4-[L2/3]. The gene expression patterns of *Wfs1* and *Cux2* were also observed in the Allen ISH database^23^ and validated by smFISH–HCR (Fig. 4a).

Second, our molecular tissue region maps bring new information to refine the anatomical CCF. For example, we identified three molecular tissue regions corresponding to the retrosplenial cortex (RSP), including CTX_A_5, CTX_A_10, and CTX_A_13. All three regions have clear marker genes and unique cell-type compositions: *Tshz2* as the pan-marker for CTX_A_5,10,13; TEGLU_10-[*Tshz2_Dkk3_Neurod6*] in CTX_A_5; TEGLU_35-[*Tshz2_Cbln1_Nrep*] in CTX_A_10; and TEGLU_30-[*Tshz2_Rxfp1_Dkk3*] in CTX_A_13 (Fig. 4b). Although these molecular tissue regions align with the anatomical RSP towards the anterior of the anterior–posterior axis (Fig. 4b, i and ii), posteriorly, they have less consensus with anatomical CCF and can potentially provide refinements to it. Specifically, posterior CTX_A_5 and 13 occupy the anatomical SUB-PRE-POST (subiculum-presubiculum-postsubiculum) region (Fig. 4b, iv and v). Furthermore, the regions defined as anatomical posterior RSP in CCF share the same molecular tissue region composition with the adjacent anatomical visual cortex (Fig. 4b, iv and v). Between the anterior and posterior parts, CTX_A_5 and 13 occupy both anatomical RSP and the anatomical SUB-PRE-POST regions (Fig. 4b, iii). Given the discrepancy between our results and the anatomical labels in CCFv3, we proceeded to confirm our molecular tissue region maps by further examining the anterior–posterior distribution of the molecular tissue region marker gene *Tshz2* in the Allen ISH database^23,26^ and by smFISH–HCR validation (Fig. 4b). Our result may provide insight into a recent related study, which identified that the anatomically defined anterior and posterior RSP showed different functions in memory formation in rodents^34^. Specifically, the inhibition of the anatomical posterior RSP selectively impaired the visual contextual memory information^34,35^, suggesting that anatomical posterior RSP defined in CCF may contain part of the adjacent visual cortex. Notably, the anatomical RSP was traditionally defined by cell and tissue morphology^36,37^ (Nissl staining or neurofilament staining) with limited gene expression information. Thus the molecular tissue regions (marked by *Tshz2*, *Cxcl14*, *Neurod6* and *Rxfp1*; Fig. 4b, Extended Data Fig. 8k) may be more accurate in delineating RSP and its subregions.

Third, we observed cases in which the joint single-cell and spatial definitions of cell types resolve cell heterogeneity better than single-cell gene expression alone. Although the dentate gyrus granule cells largely form a homogeneous cluster in the single-cell gene expression latent space, they fall into two distinct molecular tissue region clusters (CTX_HIP_1-[DGd-sg] and CTX_HIP_2-[DGv-sg]) in the spatial niche gene expression latent space, marked by enriched expression of *Epha7* and *Atp2b4*, respectively (Fig. 4c). Allen ISH database^23^ and smFISH–HCR validation confirmed the marker gene gradients along the dorsal–ventral axis (Fig. 4d). This unique molecular tissue region segmentation through spatial niche gene expression may provide insights into functional transitions along the dorsal–ventral axis of the hippocampus^26,38^.

## Transcriptome-wide gene imputation

**Fig. 5:**
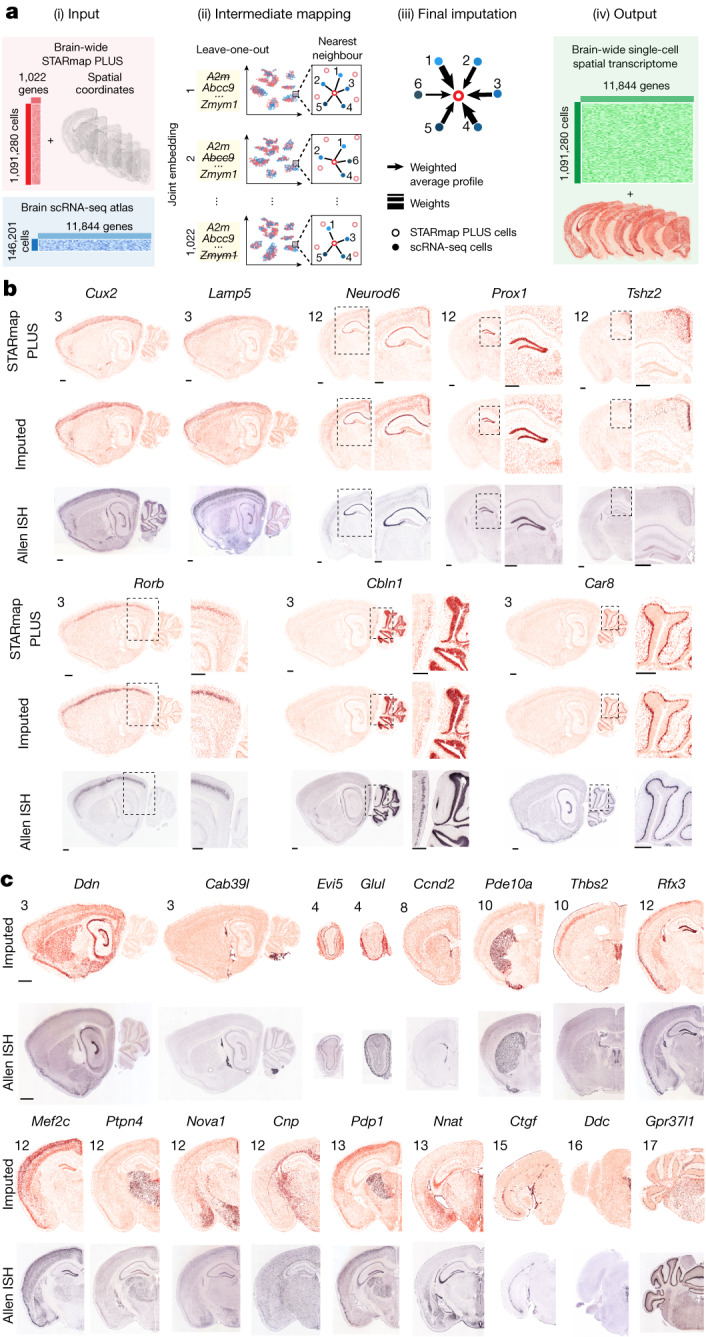
Transcriptome-scale adult mouse CNS spatial atlas by gene imputation. **a**, Schematics of the imputation workflow. Using the STARmap PLUS measurements and a scRNA-seq atlas^1^ as inputs, we first performed intermediate mappings using a leave-one-(gene)-out strategy (Methods). The resulting intermediate mappings were used to compute weights between STARmap PLUS cells and scRNA-seq cells for a final imputation to output 11,844 gene-expression profiles in STARmap PLUS cells. **b**, Representative imputed spatial gene expression maps with corresponding STARmap PLUS and Allen Mouse Brain ISH^23^ gene-expression maps. Each dot represents a cell coloured by the expression level of a gene. Scale bar, 0.5 mm. The sample slice number was labelled (top left). **c**, Examples of imputed spatial expression profile of genes outside the STARmap PLUS 1,022 gene list with the corresponding Allen ISH images^23^. Scale bar, 1 mm. The ISH data were obtained from Allen Mouse Brain Atlas^23^.

**Extended Data Fig. 9:**
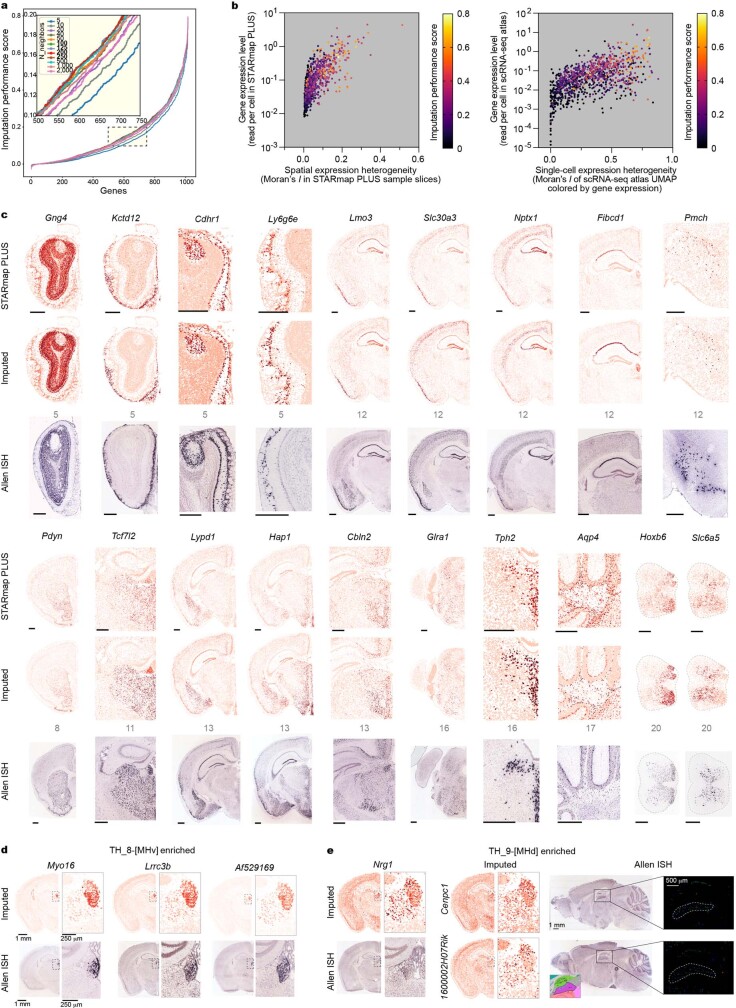
Imputation parameter optimization and performance evaluation. **a**, Cumulative curves of the imputation performance scores across STARmap PLUS genes in the intermediate mapping using different numbers of scRNA-seq cell nearest neighbors. The upper-left inset shows a zoomed-in view of the rectangular region highlighted in the bottom right. The performance score of a gene was calculated as the Pearson’s correlation coefficient (PCC, across cells) between its imputed values and measured STARmap PLUS expression level. **b**, Scatter plots of spatial expression heterogeneity (Moran’s *I*^68^ of the gene’s spatial expression map) versus gene expression level in the STARmap PLUS datasets (left), and single-cell expression heterogeneity (Moran’s *I* of scRNA-seq UMAP coloured by the gene’s expression) versus gene expression level in the scRNA-seq atlas^1^ (right). Each dot represents a gene and is coloured by the gene’s imputation performance score. *n* = 1016 genes. **c**, More examples of the comparison of imputed spatial gene expression with measured expression from STARmap PLUS and Allen Mouse Brain ISH database^23^. Each dot represents a cell coloured by the expression level of a specified gene. Scale bar, 0.5 mm. The sample slice numbers were labeled in gray. **d**,**e**, Imputed spatial gene expression heatmaps of putative marker genes of the ventral part (**d**) and the dorsal part (**e**) of medial habenula and the paired ISH images from the Allen Mouse Brain ISH database^23^. Data are provided in the accompanying Source Data file. Source data

To establish transcriptome-wide spatial profiling of the mouse CNS, we imputed single-cell transcriptomic profiles using a previously reported mutual nearest neighbours (MNN) imputation method^39^. Specifically, using 1,022-gene STARmap PLUS measurements and a scRNA-seq atlas^1^ as inputs, we generated intermediate mappings using a leave-one-(gene)-out strategy to determine the optimal nearest neighbour size (Extended Data Fig. 9a) and compute weights between STARmap PLUS cells and scRNA-seq cells for the final imputation (Methods). As a result, we imputed 11,844-gene expression profiles for 1.09 million cells in the STARmap PLUS datasets, creating a transcriptome-wide spatial cell atlas of the mouse CNS (Fig. 5a and Supplementary Table 7).

To validate the final imputation results, we compared them with measurements from the STARmap PLUS and the Allen ISH database^23^. In general, we observed higher imputation performance for genes with higher spatial and single-cell expression heterogeneity (Extended Data Fig. 9b, Supplementary Fig. 4, Supplementary Table 7 and Supplementary Discussion). For example, regional markers show consistent spatial patterns across imputed and experimental results: *Cux2* in upper cortical layers, *Rorb* in the cortical layer 4, *Prox1* in the dentate gyrus, *Tshz2* in the RSP, *Lmo3* in the piriform, *Pdyn* in the ventral striatum, *Gng4* in the olfactory bulb granule layer and *Hoxb6* and *Slc6a5* in the spinal cord (Fig. 5b and Extended Data Fig. 9c). Additionally, cell-type markers for both abundant and rare cell types were accurately imputed: cortical interneuron marker *Lamp5*, cerebellum neuron marker *Cbln1*, Purkinje cell marker *Car8* and serotonergic neuron marker *Tph2* (Fig. 5b and Extended Data Fig. 9c).

We further benchmarked the imputed results of unmeasured genes with the Allen ISH database^23^. The imputed results successfully predicted the spatial patterns of unmeasured genes (Fig. 5c), especially cell-type marker genes such as *Cab39l* (choroid plexus epithelial cells (CHOR)), *Cnp* (OLG) and *Ddc* (dopaminergic (DOP) neurons). The imputed results can also predict the relative regional expression of genes that express across multiple regions, such as *Rfx3* (a transcription factor highly expressed in dentate gyrus, PIR and choroid plexus, and modestly in cortical L2/3, dentate gyrus and ependyma), *Nova1* (an RNA-binding protein densely expressed in RSP L2/3, amygdala and medial hypothalamic nuclei, and sparsely in the LHb), and *Nnat* (a proteolipid highly expressed in the ependyma and modestly in the CA3, amygdala and medial brainstem).

Finally, we tested whether we could uncover more tissue region-specific marker genes from the imputed results. Taking the ventral medial habenula (TH_8-[MHv]) as an example, in addition to its markers in the 1,022-gene list^23,40^ (for example, *Lrrc55*, *Gm5741*, *Nwd2* and *Gng8*), the results suggest108 genes from the imputed gene list that are enriched in TH_8-[MHv] (*z*-score > 5, Supplementary Table 7), including *Af529169*, *Lrrc3b*, and *Myo16*, cross-validated with the Allen ISH database^23^ (Extended Data Fig. 9d). For the dorsal medial habenula (TH_9-[MHd]), in addition to *Wif1*, *Kcng4*, and *Pde11a*, the results suggest *Nrg1*, *Cenpc1* and *1600002H07Rik* as enriched genes (Extended Data Fig. 9e and Supplementary Table 7).

Collectively, by combining the molecular-resolution, large-scale STARmap PLUS datasets with a scRNA-seq atlas^1^, we generated a transcriptome-scale spatial single-cell expression dataset of approximately 1 million cells from the mouse CNS. This imputed, expanded atlas can be a valuable resource to discover spatially variable genes, spatially co-regulated gene programmes and cell–cell interactions.

## Quantitative AAV-PHP.eB tropism charts

**Extended Data Fig. 10:**
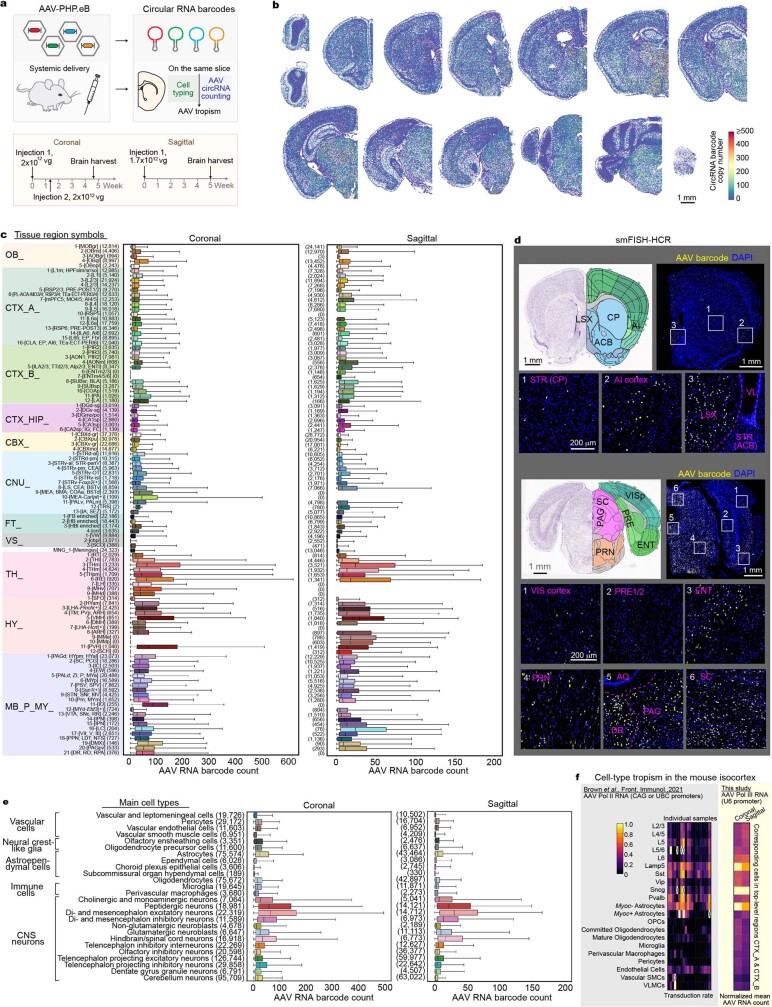
AAV barcode quantification across molecular tissue regions and molecular cell types and validation. **a**, Schematics of AAV-PHP.eB tropism characterization strategy across the adult mouse CNS. vg, viral genome. **b**, Representative spatial heatmaps showing circular RNA expression on coronal slices. Each dot represents a cell colour-coded by its AAV barcode expression level. **c**,**e**, Boxplots of circular RNA expression level across molecular tissue regions (**c**) and main molecular cell types (**e**). Boxplot elements: the vertical line, median; the box, first to third quartiles; whiskers, 2.5–97.5%. Numbers in parentheses, number of cells in the group. Abbreviations for tissue region and cell type are the same as in the main figures (also see Supplementary Tables 4 and 5). **d**, smFISH-HCR validation of AAV-PHP.eB tissue region tropisms. Images are representative of two experiments. The brain pictures were obtained from Allen Mouse Brain Atlas^18–20^. **f**, Comparison of transduction rates observed in AAV-PHP.eB tropism profiling in the mouse isocortex via scRNA-seq^44^ and the AAV RNA barcode expression in paired regions in the STARmap PLUS dataset. Anatomical tissue region abbreviations: STR, striatum; VL, lateral ventricle; LSX, lateral septal complex; CP, caudoputamen; ACB, nucleus accumbens; AI, agranular insular area; PAG, periaqueductal gray; PRN, pontine reticular nucleus; VIS, visual areas; PRE, presubiculum; ENT, entorhinal area; AQ, cerebral aqueduct; DR, dorsal nucleus raphe; SC, superior colliculus. Data are provided in the accompanying Source Data file. Source data

We further evaluated the cell-type and tissue-region tropisms of AAV, one of the leading in vivo transgene delivery tools in neuroscience research^41^. One AVV variant, PHP.eB, can efficiently cross the blood–brain barrier, enabling brain-wide gene expression^5,6^. To profile PHP.eB tropism in single cells, we combined RNA barcoding and STARmap PLUS detection, quantifying copy numbers of AAV RNA barcodes and endogenous genes in individual cells (Extended Data Fig. 10a). For optimal expression across cell types, we designed a highly expressed and stable circular RNA^15^ under a generic Pol III-transcribed U6 promoter (Extended Data Fig. 1c) rather than Pol II promoters with potential cell-type bias^42,43^. A good correlation was observed between the coronal and sagittal replicates (Pearson’s *r* ≥ 0.837, *P* < 0.0001; Supplementary Table 8), supporting the potency and robustness of our experimental and computational approaches to profiling of cell-type tropism.

Then, we assessed AAV-PHP.eB tropism across molecular tissue regions. We observed, in general, higher RNA barcode expression in the brainstem compared with the cerebrum (Extended Data Fig. 10b) and higher expression in neuron-rich regions than in glia-rich regions (for example, fibre tracts, ventricles, meninges, the choroid plexus and the subcommissural organ; Extended Data Fig. 10c). Among neuron-rich regions, thalamic molecular tissue regions show the highest transduction (Extended Data Fig. 10b,c). Using smFISH–HCR, we validated the regional preferences of PHP.eB U6 transcripts, for example, preference for the brainstem over the cerebrum and preference for the lateral septal complex over the rest of the striatum (Extended Data Fig. 10d).

Next, we examined AAV-PHP.eB tropisms across molecular cell types. We recapitulated (1) the known tropism of PHP.eB towards neurons and astrocytes^5,44^ (Extended Data Fig. 10e,f); and (2) the preference of PHP.eB^44^ for *Myoc*^−^ astrocytes (AC_1–5) over *Myoc*^+^ astrocytes (AC_6) (*P* < 0.001, unpaired one-tailed *t*-test; Supplementary Table 8). In other glial cells, OLGs, OPCs and OECs, vascular cells and immune cells show modest PHP.eB transduction. Epithelial cells (including EPEN, CHOR and subcommissural organ hypendymal cells (HYPEN)) have the lowest levels of barcode expression among all cell types (Extended Data Fig. 10e). The PHP.eB transduction profile marked by viral Pol III RNA largely aligns with the previous report^44^ using viral Pol II mRNA in the isocortex (Extended Data Fig. 10f). We further characterized PHP.eB tropism profiles among subcluster cell types (Supplementary Table 8). In summary, the mouse molecular CNS atlas offers valuable opportunities for in situ deep characterizations of viral tool tropisms.

## Discussion

This work offers a spatial molecular atlas in the mouse CNS at molecular resolution, encompassing more than 1 million cells with 1,022 genes measured by STARmap PLUS. We clustered and annotated 26 main molecular cell types, 230 subtypes, 106 molecular tissue regions and around 2,000 molecular spatial cell types jointly defined by single-cell and niche gene expression profiles in 3D space (Figs. 1–3), providing a roadmap for investigating gene-expression patterns and cell-type diagrams in the context of brain anatomy. Notably, this unbiased molecular survey of the brain enabled the discovery of new molecular cell types and tissue architectures (Fig. 4). We also expanded our 1,022 gene panel to the transcriptome scale by scRNA-seq atlas data integration and gene imputation (Fig. 5).

Our strategy and the resulting datasets have the following advantages. First, measuring RNA molecules in situ minimized the disturbance from sample preparation on single-cell expression profiles (Supplementary Discussion). Second, among spatial transcriptome mapping methods^45,46^, STARmap PLUS is unique in its high spatial resolution (200–300 nm) in all three dimensions, enabling faithful capture of 3D tissue structures with molecular gene expression information. In the future, this molecular-resolution mapping of cell transcripts and nuclear staining (Fig. 1f) could enable multimodal data analysis, such as joint cell typing by combining cell morphology and spatial transcriptomics^47^. Third, the molecular spatial profiling demonstrated here further enabled molecular tissue segmentation and data integration across different samples and technology platforms, leading to a more accurate and reproducible unified molecular definition of tissue regions compared with human-annotated anatomy. Finally, multiplexing measurements in the same sample allowed experimental integration of endogenous cellular features with exogenously introduced genetic labelling or perturbation, as illustrated here by the AAV-PHP.eB tropism profiling in the mouse CNS (Extended Data Fig. 10). This systematic strategy can be readily adapted to simultaneously profile tropisms of multiple AAV capsid variants or screen various cell-type-specific promoter and enhancer sequences within the same sample by barcoding each variant, enabling cell-type resolved, tissue-level characterization of therapeutics engagement and responses^48^.

In conclusion, we provided single-cell and spatially resolved transcriptome profiles of the mouse CNS at molecular resolution. These datasets offer potential for integration with other modalities, such as chromatin measurements, cell morphology and cell–cell communication^49^. This scalable experimental and computational framework can be readily applied to map whole-organ and whole-animal cell atlases across species and disease models, facilitating the study of development, evolution and disorders. We have complemented our atlas with an online database, mCNS_atlas with exploratory interfaces (http://brain.spatial-atlas.net) to serve as an open resource for neurobiological studies across molecular, cellular and tissue levels.

## Methods

### Plasmids

Sequences encoding the circular RNA downstream of a U6 + 27 promoter (U6 + 27-pre-racRNA) were adopted from the Tornado system (Addgene plasmid #124362)^15^ and synthesized by GenScript. Specifically, the pre-racRNA was designed to contain a unique 25-nucleotide (nt) barcode region and a shared 25-nt common sequence to enable STARmap PLUS detection (Extended Data Fig. 1c,d). The U6 + 27-pre-racRNA sequence was inserted into the vector pAAV-hSyn-mCherry (Addgene plasmid #114472) between MluI and XbaI sites, resulting in plasmid pAAV-U6-racRNA (Addgene plasmids #200824 to #200827). AAV packaging plasmids (kiCAP-AAV-PHP.eB and pHelper) were provided by the laboratory of B.E.D.

### Virus production and purification

AAV-PHP.eB expressing circular RNA barcodes were produced and purified as described^5^. In brief, pAAV-U6-racRNA and AAV packaging plasmids (kiCAP-AAV-PHP.eB and pHelper) were co-transfected into HEK 293T cells (ATCC CRL-3216) using polyethylenimine (Polysciences, 23966-1) at the ratio of 1:4:2 based on micrograms of DNA with 40 μg in total per 150-mm dish. 72 h after transfection, viral particles were collected from the medium and cells. The mixture of cells and medium was centrifuged to form cell pellets. The cell pellets were suspended in 500 mM NaCl, 40 mM Tris, 10 mM MgCl_2_, pH ~10 and 100 U ml^−1^ of salt-activated nuclease (SAN, 25 U μl^−1^, Arcticzymes, 70910-202) at 37 °C for 1 h. Viral particles from the supernatant were precipitated with 40% polyethylene glycol (Sigma, 89510-1KG-F) dissolved in 500 ml 2.5 M NaCl solution and combined with cell pellets for further incubation at 37 °C for another 30 min. Afterwards, the cell lysates were centrifuged at 2,000*g*, and the supernatant was loaded over iodixanol (Optiprep, Cosmo Bio USA, AXS-1114542) step gradients (15%, 25%, 40%, and 60%). Viruses were extracted from the 40/60% interface and the 40% layer of iodixanol gradients. Then viruses were filtered using Amicon filters (EMD, UFC910024) and formulated in sterile Dulbecco’s phosphate-buffered saline (Sigma-Aldrich, D8537). Virus titres were determined using quantitative PCR to measure the number of viral genomes (vg) after DNase I (Roche Diagnostics, 4716728001) treatment to remove the DNA not packaged and then proteinase K (Roche Diagnostics, 03115828001) treatment to digest the viral capsid and expose the viral genome. Quantified linearized plasmids of pAAV-U6-racRNA were used as a DNA standard to transform the *C*_t_ value to the amount of viral genome. The virus titre of AAV-PHP.eB.1 (barcode set 1) for coronal samples: 2 × 10^13^ vg ml^−1^; AAV-PHP.eB.2 (barcode set 2) for sagittal samples: 1.7 × 10^13^ vg ml^−1^.

### Mice and tissue preparation

The following mice were used in this study: C57BL/6 (Charles River Laboratories, strain code: 475, female, 8–10 weeks old) and B6.Cg-Tg(Thy1-YFP)HJrs/J (Jackson Laboratory, 003782, male, 5 weeks old). Mice were housed 2–5 per cage and kept on a 12-h light–dark cycle with ad libitum food and water at the temperature of 65–75 °F (approximately 18–23 °C) with 40–60% humidity. For virus injection, mice were anaesthetized with isoflurane (3–5% induction, 1–2% maintaining). We sampled mouse CNS tissues at least four weeks post-injection, when viral responses were shown to return to the control level to minimize the side effect of AAV infection on cell typing^44^. Experimental procedures were approved by the Institutional Animal Care and Use Committee (IACUC) of the Broad Institute of MIT and Harvard under animal protocol no. 0255-08-19. Detailed information regarding mice and tissue sample slices was included in Supplementary Table 3.

#### Mouse brain and spinal cord coronal sections

Intravenous administration of AAV-PHP.eB.1 at 2 × 10^12^ vg was performed by injection into the retro-orbital sinus of adult mice (C57BL/6, female, 8–10 weeks of age). One week after the first injection, a second injection was administered to enhance expression. Thirty days after the first injection, mice were anaesthetized with isoflurane (Extended Data Fig. 10a). The brain tissue was collected after rapid decapitation. The spinal cord was isolated using hydraulic extrusion to reduce handling time and the risk of damage to the tissue^51^. In brief, the large end of a 200-μl non-filter pipette tip was trimmed and fit firmly onto a 5-ml syringe. Next, the spinal column was cut on both sides past the pelvic bone through the rostral-caudal axis, straightening and trimming at both proximal- and distal-most ends until the spinal cord was visible. A 5-ml syringe filled with ice-cold PBS (Gibco, 10010049) was inserted at the distal-most end of the spinal column, and steady pressure was applied to extrude the spinal cord into a 100-mm Petri dish filled with sterile PBS on ice. The lumbar segments of the spinal cord tissue were collected. Tissues were placed in OCT (Fisher, 23-730-571), frozen in liquid nitrogen, and sliced into 20-μm sections using a cryostat (Leica CM1950) at −20 °C.

#### Mouse brain sagittal sections

Intravenous administration of AAV-PHP.eB.2 at 1.7 × 10^12^ vg was performed by injection into the retro-orbital sinus of an adult Thy1-EYFP mouse (B6.Cg-Tg(Thy1-YFP)HJrs/J, male, five weeks of age). After five weeks of expression, mice were anaesthetized with isoflurane and transcardially perfused with 50 ml ice-cold DPBS (Sigma-Aldrich, D8537) (Extended Data Fig. 10a). The brain tissue was then removed, placed in OCT, frozen in liquid nitrogen, and sliced into 20-μm sagittal sections using a cryostat (Leica CM1950) at −20 °C.

### 1,022-gene list selection and STARmap PLUS probe design

Cell-type marker genes and most differentially expressed genes were extracted from scRNA-seq studies that systematically surveyed the adult mouse CNS, which included multiple brain regions from the forebrain to the hindbrain and sampled the cells with minimum selection^1,2^. The list was further supplemented with the Allen Mouse Brain transcriptome database markers^13^. The list was curated to 1,022 genes to be uniquely encoded by 5-digit identifiers (Extended Data Fig. 1a and Supplementary Table 1).

STARmap PLUS probes for the 1,022 genes were designed as previously described with modifications to further improve the specificity of target transcript detection^3,4^. The backbone of padlock probes contains a 5-nt gene-specific identifier and a universal region where reading probes align (Extended Data Fig. 1b). In addition, a second 3-nt barcode was introduced to the DNA–DNA hybridization region between a pair of primer and padlock probes to reduce the possibility of false positives caused by intermolecular proximity where the primer for transcript identity *A* leads to circularization of the padlock hybridized to transcript identity *B*. For the SEDAL step, the homemade sequencing reagents included six reading probes (R1 to R6) and 16 two-base encoding fluorescent probes (2base_F1 to 2base_F16) labelled with Alexa 488, 546, 594, and 647 (Supplementary Table 2).

To detect RNA barcodes, a primer was designed to hybridize to the common 25-nt region while a pool of padlock probes was designed to hybridize to variable 25-nt barcode region, converting the barcode into a barcode-unique identifier (Extended Data Fig. 1d). This identifier was sequenced in one round of SEDAL by an orthogonal reading probe (R7 for coronal samples and R8 for sagittal samples) and four one-base encoding fluorescent probes (1base_F1 to 1base_F4) labelled with Alexa 488, 546, 594 and 647 (Supplementary Table 2).

### Publicly available data

Publicly available data used in this study: Allen Mouse Brain Reference Atlas^18–20^ (ISH^23^, CCFv3 (ref. ^20^)); scRNA-seq datasets of adult mouse nervous system^1^ (available at Sequence Read Archive (https://www.ncbi.nlm.nih.gov/sra) under accession SRP135960), cerebellum^25^ (available at Gene Expression Omnibus (GEO) under accession number GSE165371), striatum^10^ (available at GEO under accession number GSE118020), and whole cortex and hippocampus^26^ (in the NeMO Archive for the BRAIN Initiative Cell Census Network: https://assets.nemoarchive.org/dat-jb2f34y); and processed AAV-PHP.eB transduction rate in cortical cell types (available at CaltechData: 10.22002/D1.2090, aavomics_cell_type_transduction_rates.csv)^44^.

### Software

The following packages and software^50,52–62^ were used in the data analysis: ClusterMap is implemented based on MATLAB R2019b and Python 3.6. The following packages and software were used in data analysis: UCSF ChimeraX 1.0, ImageJ 1.51, MATLAB R2019b, R 4.0.4, RStudio 1.4.1106, Jupyter Notebook 6.0.3, Anaconda 2-2-.02, h5py 3.1.0, hdbscan 0.8.36, hdf5 1.10.4, matplotlib 3.1.3, seaborn 0.11.0, scanpy 1.6.0, numpy 1.19.4, scipy 1.6.3, pandas 1.2.3, scikit-learn 0.22, umap-learn0.4.3, pip 21.0.1, numba 0.51.2, tifffile 2020.10.1, scikit-image 0.18.1, squidpy 1.1.2, anndata 0.8.0 and itertools 8.0.0.

### STARmap PLUS

The STARmap PLUS procedure was performed as previously described^3,4^ with minor modifications.

#### Sample preparation

Glass-bottom 6- or 12-well plates (MatTek, P06G-1.5-20-F and P12G-1.5-14-F) were treated with methacryloxypropyltrimethoxysilane (Bind-Silane, GE Healthcare, 17-1330-01), followed by a poly-d-lysine solution (Sigma A-003-E). No. 2 Micro cover glasses (12 mm or 18 mm, Electron Microscopy Sciences, 72226-01 or 72256-03) were pretreated with Gel Slick solution (Lonza, 50640) following the manufacturer’s instructions for later polymerization. 20-μm coronal and sagittal slices were mounted in the pretreated glass-bottom 12-well and 6-well plates, respectively. Tissue slices were fixed with 4% PFA (Electron Microscopy Sciences, 15710-S) in PBS at room temperature for 10 min, permeabilized with pre-chilled methanol (Sigma-Aldrich, 34860-1L-R) at −80 °C for 30 min, and re-hydrated with PBSTR/glycine/YtRNA (PBS with 0.1% Tween-20 (Teknova, 100216-360), 0.1 U µl^−1^ SUPERase-In (Invitrogen, AM2696), 100 mM glycine (VWR, M103-1KG), 0.1 mg ml^−1^ yeast tRNA (Invitrogen, AM7119)) at room temperature for 15 min before hybridization. For sagittal slices, the step of methanol treatment was skipped, and the sample was permeabilized with 1% Triton X-100 (Sigma-Aldrich, 93443) in PBS with 0.1 U µl^−1^ SUPERaseIn, 100 mM glycine, and 1% yeast tRNA at room temperature for 15 min.

#### Library construction

The reaction volumes listed below were for 12-well plate wells. For 6-well plate wells, the reaction volume was doubled. Stock SNAIL probes were dissolved to 50 nM or 100 nM per probe in IDTE pH 7.5 buffer (IDT, 11-01-02-02). The final concentration per probe for hybridization was as follows: SNAIL probes for mouse 1,022 genes, 5 nM; primers for RNA barcodes, 100 nM; padlock probes for RNA barcodes, 10 nM for coronal samples, and 100 nM for sagittal samples. The brain slices were incubated in 300 µl hybridization buffer (2× SSC (Sigma-Aldrich, S6639), 10% formamide (Calbiochem, 344206), 1% Triton X-100, 20 mM ribonucleoside vanadyl complex (New England Biolabs, S1402S), 0.1 mg ml^−1^ yeast tRNA, 0.1 U µl^−1^ SUPERaseIn, and SNAIL probes) at 40 °C for 24–36 h with gentle shaking.

The samples were then washed at 37 °C for 20 min with 600 µl PBSTR (PBS, 0.1% Tween-20, 0.1 U µl^−1^ SUPERaseIn) twice, followed by one wash at 37 °C for 20 min with 600 µl high salt buffer (PBSTR, 4× SSC). After a brief rinse with PBSTR at room temperature, the samples were then incubated for 2 h with a 300 µl T4 DNA ligase mixture (0.1 U µl^−1^ T4 DNA ligase (Thermo Scientific, EL0011), 1× T4 ligase buffer, 0.2 mg ml^−1^ BSA (New England Biolabs, B9000S), 0.2 U µl^−1^ of SUPERase-In) at room temperature with gentle shaking, followed by 2 washes with 600 µl PBSTR. Then the sample was incubated with 300 µl rolling-circle amplification mixture (0.2 U µl^−1^ Phi29 DNA polymerase (Thermo Scientific, EP0094), 1× Phi29 reaction buffer, 250 µM dNTP mixture (New England Biolabs, N0447S), 0.2 mg ml^−1^ BSA, 0.2 U µl^−1^ of SUPERase-In and 20 µM 5-(3-aminoallyl)-dUTP (Invitrogen, AM8439)) at 4 °C for 30 min for equilibrium and then at 30 °C for 2 h for amplification.

The samples were next washed twice in 600 µl PBST (PBS, 0.1% Tween-20) and treated with 400 µl 20 mM acrylic acid NHS ester (Sigma-Aldrich, 730300) in 100 mM NaHCO_3_ (pH 8.0) for 1 h at room temperature. The samples were washed briefly with 600 µl PBST once, then incubated with 400 µl monomer buffer (4% acrylamide (Bio-Rad, 161-0140), 0.2% bis-acrylamide (Bio-Rad, 161-0142), 2× SSC) for 30 min at room temperature. The buffer was removed, and 25 µl of polymerization mixture (0.2% ammonium persulfate (Sigma-Aldrich, A3678), 0.2% tetramethylethylenediamine (Sigma-Aldrich, T9281) in monomer buffer) was added to the centre of the sample, which was immediately covered by Gel Slick coated coverslip and incubated for 1 h at room temperature under nitrogen gas atmosphere. The samples were then washed with 600 µl PBST twice for 5 min each. Except for sagittal brain slices, the tissue-gel hybrids were digested with Proteinase K (Invitrogen, 25530049, 0.2 mg ml^−1^ in 50 mM Tris-HCl 8.0, 100 mM NaCl, 1% SDS (Calbiochem, 7991)) at room temperature overnight, then washed with 600 µl 1 mM AEBSF (Sigma-Aldrich, 101500) in PBST once at room temperature for 5 min and another two washes with PBST. Samples were stored in PBST at 4 °C until imaging and sequencing.

#### Imaging and sequencing

Before SEDAL, the samples were washed twice with the stripping buffer (60% formamide and 0.1% Triton X-100 in water) and treated with the dephosphorylation mixture (0.25 U µl^−1^ Antarctic Phosphatase (New England Biolabs, M0289L), 1× reaction buffer, 0.2 mg ml^−1^ BSA) at 37 °C for 1 h. Each cycle of SEDAL began with two washes with the stripping buffer (10 min each) and three washes with PBST (5 min each). For the six-round of 1,022-gene SEDAL, the sample was incubated with the ‘sequencing by ligation’ mixture (0.2 U µl^−1^ T4 DNA ligase, 1× T4 DNA ligase buffer, 0.2 mg ml^−1^ BSA, 10 µM reading probe, and 300 nM of each of the 16 two-base encoding fluorescent probes) at room temperature for 3 h. For the round of RNA barcode SEDAL, the sample was incubated with (0.1 U µl^−1^ T4 DNA ligase, 1× T4 DNA ligase buffer, 0.2 mg ml^−1^ BSA, 5 µM reading probe, 100 nM of each of the 4 one-base fluorescent oligos) at room temperature for 1 h. After three washes with the wash and imaging buffer (10% formamide, 2× SSC in water, 10 min each) and DAPI staining (Invitrogen, D1306, 100 ng ml^−1^), the sample was imaged in the wash and imaging buffer.

Images were acquired using Leica TCS SP8 or Stellaris 8 confocal microscope using LAS X software (SP8: version 3.5.5.19976; Stellaris 8: version 4.4.0.24861) with a 405 nm diode, a white light laser, and 40× oil immersion objective (NA 1.3) with a voxel size of 194 nm × 194 nm × 345 nm. DAPI was imaged at the first round of 1,022-gene SEDAL and the round of RNA barcode SEDAL to enable image registration (Extended Data Fig. 2a).

### STARmap PLUS data processing

#### Pre-processing, deconvolution, registration and spot-calling

Image deconvolution was achieved with Huygens Essential version 21.04 (Scientific Volume Imaging), using the classic maximum likelihood estimation method, with a signal to noise ratio of 10 and 10 iterations. Image registration, spot calling and barcode filtering were applied according to previous reports^3,4^.

#### ClusterMap cell segmentation

We applied ClusterMap^12^ method to segment cells by amplicons (mRNA spots) with quality control for gene spots and pre- and post-processing. First, a background identification process was used to filter input spots. Specifically, 10% of local low-density mRNA spots were considered as background noises and were removed before the downstream analysis. Second, an additional step of noise rejection was used after mRNA spot clustering as post-processing. Specifically, we removed cells that do not overlap with DAPI signals. These quality control steps for mRNA spots have been included in the analysis of all 20 coronal and sagittal datasets.

#### Quality control for cells

First, we excluded low-quality cells with standard preprocessing procedures in Scanpy^63^. Here we combined and analysed 20 coronal and sagittal datasets together. We set the minimum gene number per cell and minimum cell number per gene as 20, the minimum read count per cell as 30, and the maximum read count per cell as 1,300. After filtering, we obtained a data matrix of 1,099,408 cells by 1,022 genes. Then the matrix was normalized across each cell and logarithmically transformed. The effects of total read count per cell were regressed out and the data was finally scaled to unit variance.

#### Batch effect evaluation and correction

To evaluate batch effects, we grouped adjacent tissue slices into batches. We checked batch effects across labelled batch samples A–J (Supplementary Table 3). We first observed and corrected the batch effect between coronal samples in groups C and D using Combat^64^. We also observed and corrected the batch effect between coronal and sagittal samples. The function scanpy.pp.combat was used for batch effect correction.

### Cell-type annotations

#### Integration with scRNA-seq dataset

We first used Harmony^16^ to integrate STARmap PLUS datasets and a scRNA-seq dataset^1^ of the mouse nervous system. We used the overlapped 1,021 genes between the STARmap PLUS and the scRNA-seq datasets to compute adjusted principal components and performed joint clustering to transfer main-level cell-type labels in the scRNA-seq dataset^1^ to STARmap PLUS identified cells. The function scanpy.external.pp.harmony_integrate was used to perform the integration. The function scanpy.tl.leiden was used with a resolution equal to 1 to perform joint clustering.

#### Main cluster and subcluster cell-type annotation

The main-level clustering and annotation of STARmap PLUS identified cells were decided based on integration of STARmap PLUS datasets with the public scRNA-seq dataset^1^.

First, as discussed above, we integrated STARmap PLUS cells with cells in the scRNA-seq dataset. Second, we performed joint Leiden clustering on all integrated cells, recovering 53 joint clusters. Third, we transferred labels of cells in scRNA-seq datasets with principle described as follows. Within each joint cluster, we checked the cell-type labels of scRNA-seq cells. If the number of top-1 scRNA-seq cell-type labels within one joint cluster exceeded 80%, it indicated successful integration of multi-source single-cell datasets on this cell type. Therefore, we assigned this dominant top-1 scRNA-seq cell-type label to STARmap PLUS cells in that joint cluster with high confidence. Otherwise, we regarded integration as unsuccessful and temporarily labelled the joint cluster as ‘NA’. We annotated STARmap PLUS cells at four levels with this principle using rank 1 to rank 4 cell-type labels in the scRNA-seq dataset. A higher rank means more detailed annotation. Specifically, we annotated cells into 4 cell types at rank 1 level; 5 cell types at rank 2 level, 13 cell types at rank 3 level, and 22 cell types at rank 4 level. There existed a portion of cells as NA types in levels of rank 2 to rank 4. Finally, the rank 4 level annotation was defined as the main-level annotation (main cell types).

We then investigated individual main cell types and manually annotated detailed sublevel cell types (Supplementary Figs. 2 and 3). First, we extracted cells in each main-level cluster and performed Leiden clustering to determine subclusters. Specifically, we excluded genes with either a maximum read count per cell of less than 10 or with expression detected in fewer than 10 cells at a count threshold of 5, computed principal component analysis (PCA) and UMAP, and performed Leiden clustering on the *k*NN constructed on the principle component space. Functions scanpy.tl.pca, scanpy.pp.neighbors, scanpy.tl.umap and scanpy.tl.leiden were used.

Second, we manually annotated each subclusters based on marker genes and spatial cell distribution (Supplementary Table 4). Specifically, we first identified the top five marker genes for each subcluster using scanpy.tl.rank_genes_groups. In each subcluster, we checked the dot plot showing the fraction of cells expressing specific marker genes and the mean expression of specific marker genes. The marker genes highly expressed across multiple cell types are recognized as common markers. The markers with specific expressions in a particular subcluster are identified as cluster-specific markers. In addition, we examined and confirmed those marker genes in other scRNA-seq databases^1,2,26^. We refined the marker gene list as described above and annotated the subclusters with the most relevant cell types based on the remaining marker genes. Second, to narrow down to a unique annotation or distinguish subclusters with same annotations, we checked the spatial cell distribution of each subcluster. We observed that some subclusters were explicitly distributed in certain brain regions, allowing us to rule out irrelevant candidates. As for the remaining undetermined subclusters, we combined them with the most relevant annotated subclusters or split them further using Leiden clustering based on prior knowledge.

Third, we analysed cells in the NA cluster, assigning these cells to valid cell types and combining them into rank 4 clusters when appropriate. Specifically, the following types were recovered from the rank 4 NA cells: HYPEN; non-glutamatergic neuroblasts (NGNBL); cerebellar Purkinje cells (CBPC, combined into rank 4 cerebellum neurons); *Th*^+^ OBINH (OBINH_7, combined into rank 4 OBINH neurons). Additionally, vascular-like cells in the NA cluster were combined with rank 4 vascular cells and re-clustered. Neuronal-like cells in the NA cluster were combined with rank 4 DE/MEINH and rank 4 hindbrain neurons and re-clustered (Supplementary Fig. 2k). There remain 12 unannotated subclusters (1.8% of total cells) due to lack of annotatable marker genes (Supplementary Fig. 2n), which may have resulted from the differences in sampling coverage between the scRNA-seq and STARmap PLUS datasets.

It is worth mentioning that the cell-typing results in this study were based on the consensus between the STARmap PLUS dataset and published scRNA-seq datasets, followed by manual annotation. The STARmap PLUS dataset mapped more cells than the previous scRNA-seq dataset^1^, potentiating more detailed cell typing and annotations in the future.

A schematic summary of the cell typing workflow is shown in Extended Data Fig. 2c.

### Near-range cell–cell adjacency analysis

We quantified the number of edges between cells of each main cell type with cells of other main cell types as previously described^12,65^. In brief, we first constructed a mesh graph by Delaunay triangulation of cells in each sample using squidpy.gr.spatial_neighbors. Then we computed a near-range cell–cell adjacency matrix from spatial connectivity using squidpy.gr.interaction_matrix. We normalized the matrix along the row and column axes sequentially as shown in Extended Data Fig. 4g. A similar analysis was performed at the subcluster cell type level and reported in Supplementary Table 4.

### Molecular tissue region analysis

#### Molecular tissue region clustering based on spatial niche gene expression

For a given sample, the smoothed expression vector of each cell was represented by concatenating that of its *k* nearest spatial neighbours, including itself^66^. The spatially smoothed-expression matrices for each sample were then stacked into a single dataset and passed into the PCA followed by Harmony^16^ for integration. Clustering was then performed in the principal component space using the Leiden algorithm followed by visualization using UMAP^50^.

The value *k* was set to 30 neighbours for the identification of broad anatomical regions (level 1), such as the neocortex. To identify subregions (level 2), such as individual neocortical layers, subclustering of each level 1 region was performed with varying *k* values depending on the morphology of expected subregions (Supplementary Table 5). For example, as meninges are inherently thin, we expected subregions of meninges to also be thin and thus require a smaller neighbourhood size *k* in order to avoid smoothing away their finer structure. A final level of clustering was then applied to a subset of level 2 regions to identify more subregions (level 3) that were expected based on manual inspection of level 2 gene markers.

Note that, for a certain sample slice, when the number of cells in a cluster is smaller than the value *k* for smoothing, the concatenated spatial niche gene expression vector cannot be made. In this case, the cell is rejected from further subclustering. To take care of those rejected cells, we performed post-processing to transfer tissue region labels from their physical neighbouring cells (see below).

A resolution parameter must also be specified for each instance of clustering. Resolutions for each level of clustering were manually tuned (Supplementary Table 5) to capture known anatomical features based on the Allen Institute Mouse Atlas as well as preliminary marker genes calculated using differentially expressed gene analysis via the rank_genes_groups function in Scanpy^63^.

To identify tissue region marker genes, we first calculated the average expression of each gene across all the cells of each region. Then for each gene, its percentage distribution across tissue regions was normalized to *z*-scores (Supplementary Table 5).

Finally, we manually combined fragmented subclusters originating from different main clusters when appropriate. To guide manual curation of spatial clustering, non-negative matrix factorization^67^ was applied to the stacked and spatially smoothed expression matrix (that is, the matrix passed into PCA/Harmony above), identifying anatomical factors along with corresponding gene factor loadings.

#### Molecular tissue region label post-processing

We first assigned tissue region labels for those cells missing annotation. Under level 1 tissue region labels, we performed the *k*NNs (here *k* = 5) classification to assign a level-1 tissue region label for those cells missing level 1 annotation. Similarly, under level 2 and level 3 tissue region labels, respectively, we performed the *k*NNs (here *k* = 5) classification to assign a level 2 or level 3 tissue region label for those cells missing level 2 or level 3 annotation.

We then performed smoothing based on level 3 tissue region labels (*k*NNs, here *k* = 50), and we manually adjusted some molecular tissue region labels as listed below. First, cells in the ‘meninges’ molecular tissue regions were excluded from the smoothing process to minimize the effect on the nearby tissue regions. Second, we observed that cell-sparse regions (for example, molecular layers) would be overwhelmed by a nearby cell-dense region (for example, granule cell regions) during this smoothing process. Therefore, we manually kept the molecular tissue region cluster labels unchanged for those cells (including OB_5-[OBopl] and CTX_HIP_3-[DGmo/po]).

#### Allen Mouse Brain Common Coordinate Framework (CCFv3) registration, label transfer and molecular tissue region annotation

We performed registration of each STARmap PLUS tissue slice with Allen CCFv3 (ref. ^20^) according to public resources^21,22^. The molecular cell-type maps of STARmap PLUS slices were used to facilitate registration. Specifically, we first manually extracted one corresponding slice image from Allen CCFv3. Next, we manually clicked paired anchors in the STARmap PLUS slice and the corresponding Allen CCFv3 slice for registration. The package AP_histology^21^ provided the analysis above.

After registration, we had a paired Allen CCFv3 slice for each of our STARmap PLUS tissue slices. We then applied an inverse transformation to the paired Allen CCFv3 slices and assigned labels of Allen CCF anatomical regions to cells in STARmap PLUS tissue slices to facilitate molecular tissue region annotation.

### RNA Hybridization Chain Reaction (HCR)

We performed smFISH–HCR (v3.0)^24^ on thin brain tissue slices (20 µm) using commercial HCR buffers and HCR amplifiers according to the manufacturer’s instructions (Molecular Instruments). C57BL/6 mice (Jackson Laboratory, 000664, male, 10–13 weeks old) were used in the smFISH–HCR validation experiments. In brief, tissue slices were fixed with 4% PFA in PBS on ice for 15 min, permeabilized with ice-cold methanol for 30 min, and washed with PBSTR (PBS, 0.1% Tween-20, 0.1 U µl^−1^ SUPERase-In) twice at room temperature for 10 min. The sample was then pre-incubated in the HCR probe hybridization buffer at 37 °C for 10 min and then incubated at 37 °C for 12–16 h with custom-designed three or four pairs of HCR probes (final concentration of 25–100 nM for each probe) in the HCR probe hybridization buffer supplemented with 0.1 mg ml^−1^ Yeast tRNA and 0.1 U µl^−1^ SUPERase-In. The day after, the sample was washed with the HCR probe wash buffer, and the signal was amplified with the HCR amplifier probes at room temperature for 8–16 h. The fluorescent amplification probe sets used included B1-Alexa647, B2-Alexa594, B3-Alexa546, and B5-Alexa488. Finally, the sample was washed with 5×SSCT (5×SSC, 0.1% Tween-20), stained with DAPI, and imaged in PBS with 10% SlowFade Gold Antifade Mountant with DAPI (Invitrogen, S36938) with Leica Stellaris 8 confocal microscope. Sequence information for HCR probes is available in Supplementary Table 2.

### Imputation

We performed imputation of unmeasured genes after integrating the scRNA-seq dataset and STARmap PLUS dataset, following a similar imputation strategy as described^39^.

First, we performed intermediate mapping. Specifically, for each of the 1,022 genes in the STARmap PLUS, we left one gene out and performed an intermediate mapping to align each STARmap PLUS cell with the most similar set of cells in the scRNA-seq dataset. The dimension reduction and batch effect correction methods were PCA, UMAP and Harmony (the same as the previous analysis). Here, the leave-one-(gene)-out mapping approach was used to assess the performance changes caused by varying the number of nearest neighbours in scRNA-seq data. We evaluated the performance score for each mapped gene. The performance score was calculated as the Pearson’s correlation *r* (across cells) between its imputed values and measured STARmap PLUS expression level. According to the result in Extended Data Fig. 9a, we chose the number of nearest neighbours to be 200.

Finally, we performed a final imputation. We first generated imputation gene list based on the scRNA-seq data^1^: genes with average read <0.005 (that is, sum read <740 across 146,201 cells, 50th percentile of the data) were filtered; genes with maximum read ≤10 were filtered. This resulted in 11,844 genes after the filtration and we used those genes for imputation. To perform imputation for all genes, we aggregated across the intermediate mappings generated from each gene probed by STARmap PLUS. Specifically, for each STARmap PLUS cell, we considered the set of all scRNA-seq cells that were associated with it in any intermediate mapping. Subsequently, for every cell, we calculated each gene’s imputed expression level as the weighted average of the gene’s expression across the associated set of scRNA-seq cells, where weights were proportional to the number of times each scRNA-seq cell was present (Fig. 5a). Thus, the imputed expression profiles for all genes, including those in the overlapping gene set, are on the same scale as the scRNA-seq log count data. The output is a 1,091,280-cell by 11,844-gene matrix. We also evaluated the performance score for the imputed genes by comparing them to Allen ISH data^23^. Representative results are shown in Fig. 5b and Extended Data Fig. 9c.

Using the genes with STARmap PLUS measured ground-truth, we examined the following gene expression features for their association with the imputation performance in the leave-one-(gene)-out intermediate imputation (Extended Data Fig. 9b, Supplementary Discussion and Supplementary Fig. 4). (1) Gene expression level in STARmap PLUS. (2) Spatial expression heterogeneity in STARmap PLUS. For each gene, Moran’s *I* (a coefficient measuring overall spatial autocorrelation^68^) for the gene’s spatial expression was calculated for each of the 20 sample slices by a function squidpy.gr.spatial_autocorr^65^ and then averaged, to represent the degree of patterned spatial expression. Higher Moran’s *I* represents more patterned spatial gene expression. (3) Gene expression in scRNA-seq dataset^1^. (4) Single-cell expression heterogeneity in scRNA-seq dataset^1^. We quantified the degree of cell expression specificity of a gene by calculating Moran’s *I* of the scRNA-seq^1^ UMAP coloured by the gene’s expression.

### Trajectory analysis

OLGs and OPCs were explored for their developmental trajectory. These cells have subcluster annotations as OLG_1, OLG_2, OLG_3, and OPC, following the analysis described in ‘Main cluster and subcluster cell-type annotation’.

To quantify developmental stages, we computed PCA, neighbours (*k*NN graph) and diffusion maps using functions scanpy.tl.pca, scanpy.pp.neighbors and scanpy.tl.diffmap. The Scanpy package was utilized for diffusion map^63,69^.

### Cell-type cluster correspondence with brain subregion scRNA-seq datasets

We integrated our STARmap PLUS data of specific regions with existing regional scRNA-seq datasets to examine the cross-dataset nomenclature correspondence for cell types.

We first referred to a scRNA-seq dataset in the mouse brain cortex and hippocampus (https://portal.brain-map.org/atlases-and-data/rnaseq)^26^. We extracted STARmap PLUS cells labelled in top-level molecular tissue regions CTX_A, CTX_B, L1_HPFmo_MNG, CTX_HIP_CA, CTX_HIP_DG and ENTm. For integration of these STARmap PLUS cells and the scRNA-seq dataset, we performed similar analyses as described in ‘Cell-type annotations’. We first used Harmony^16^ to integrate all cells. Then we used the overlapped genes between STARmap PLUS and scRNA-seq experiments to compute adjusted principal components and performed joint clustering to transfer cell-type labels in the scRNA-seq dataset to STARmap PLUS cells. The transferred labels for STARmap PLUS cells were decided based on the integration of STARmap PLUS cells with the scRNA-seq dataset. Within each joint cluster, we checked the cell-type labels of those scRNA-seq cells. If the number of top-1 scRNA-seq cell-type labels within one joint cluster exceeded 60%, it indicated successful integration for multi-source single-cell datasets on this cell type. Therefore, we assigned this dominant top-1 scRNA-seq cell-type label to STARmap PLUS cells in that joint cluster with high confidence. Otherwise, we regarded integration as unsuccessful and did not transfer labels from the scRNA-seq dataset to STARmap PLUS cells. The function scanpy.external.pp.harmony_integrate was used to perform the integration.

Then, similarly, we referred to a scRNA-seq dataset in mouse brain striatum^10^ and a scRNA-seq dataset in mouse cerebellum^25^ and performed the same analysis to generate correspondence for cell types. For the striatum, we extracted cells labelled as top-level molecular tissue region ‘STR’. For the cerebellum, we extracted cells labelled as top-level molecular tissue regions CBX_1 and CBX_2.

### RNA barcode analysis

#### Assigning circular RNA barcode spots into cells

We first performed spot-calling of circular RNA barcode spots, according to the same process as that in ‘STARmap PLUS data processing’. Then, in each tile, we binarized the DAPI signal and used it as a mask to remove circular RNA barcode reads outside the cell nucleus. Then we stitched the spots in each tile together based on tile location information. We next assigned circular RNA barcode spots into cells identified by endogenous genes. Using sklearn.neighbors.NearestNeighbors (*k* = 1), we located the nearest mRNA spot to each circular RNA barcode spot. We then associated the cell identity of the mRNA spot with the circular RNA barcode amplicon. Finally, we counted the total number of circular RNA barcodes for each cell.

#### Cell-type-based statistics

For each main and subtype cell cluster, we computed summary statistics of the 2.5th, 25th, 50th, 75th and 97.5th percentiles using numpy.quantile to generate a box plot of circular RNA barcode expression by cell type in both coronal and sagittal samples (Supplementary Table 8).

#### Tissue region-based statistics

We similarly computed the 2.5th, 25th, 50th, 75th and 97.5th percentiles for each tissue region after grouping cells by the tissue regions as generated above (Supplementary Table 8).

### Statistical analysis

Spearman’s *r* and its *P* values (two-tailed) in Supplementary Fig. 1 and Pearson’s *r* and its *P* values (two-tailed) in Supplementary Discussion were calculated with GraphPad Prism Version 9.3.1. *P* values in Supplementary Fig. 4 were calculated with two-sided Mann–Whitney–Wilcoxon tests by statannotations (version 0.4.4) using the function statannotations.Annotator.annotator.configure (test = ‘Mann-Whitney’, text_format = ‘star’, loc = ‘outside’). **P < 0.01, ***P < 0.001, ****P < 0.0001.

### Reporting summary

Further information on research design is available in the Nature Portfolio Reporting Summary linked to this article.

## Online content

Any methods, additional references, Nature Portfolio reporting summaries, source data, extended data, supplementary information, acknowledgements, peer review information; details of author contributions and competing interests; and statements of data and code availability are available at 10.1038/s41586-023-06569-5.

## Supplementary information

Supplementary Information

Reporting Summary

Supplementary Data This file contains source data for supplementary figure 1.

Supplementary Table 1

Supplementary Table 2

Supplementary Table 3

Supplementary Table 4

Supplementary Table 5

Supplementary Table 6

Supplementary Table 7

Supplementary Table 8

## Source data

Source Data Fig. 3

Source Data Extended Data Fig. 2

Source Data Extended Data Fig. 4

Source Data Extended Data Fig. 5

Source Data Extended Data Fig. 7

Source Data Extended Data Fig. 8

Source Data Extended Data Fig. 9

Source Data Extended Data Fig. 10

## Data Availability

The STARmap PLUS sequencing data of this study are available on the Single Cell Portal (https://singlecell.broadinstitute.org/single_cell/study/SCP1830) and Zenodo (10.5281/zenodo.8327576). We also introduced an interactive online database (http://brain.spatial-atlas.net) for exploratory analysis and hypothesis generation. Source data are provided with this paper.

## Code availability

The code that supports the analyses in this study is available at https://github.com/wanglab-broad/mCNS-atlas.
