# Publisher Correction: Spatial atlas of the mouse central nervous system at molecular resolution

**DOI:** 10.1038/s41586-023-06920-w

**Published:** 2023-12-08

**Authors:** Hailing Shi, Yichun He, Yiming Zhou, Jiahao Huang, Kamal Maher, Brandon Wang, Zefang Tang, Shuchen Luo, Peng Tan, Morgan Wu, Zuwan Lin, Jingyi Ren, Yaman Thapa, Xin Tang, Ken Y. Chan, Benjamin E. Deverman, Hao Shen, Albert Liu, Jia Liu, Xiao Wang

**Affiliations:** 1 Broad Institute of MIT and Harvard, Cambridge, MA USA; 2 Department of Chemistry, Massachusetts Institute of Technology, Cambridge, MA USA; 3 John A. Paulson School of Engineering and Applied Sciences, Harvard University, Boston, MA USA; 4 Computational and Systems Biology PhD Program, Massachusetts Institute of Technology, Cambridge, MA USA; 5 Department of Electrical Engineering and Computer Science, Massachusetts Institute of Technology, Cambridge, MA USA; 6 Department of Biology, Massachusetts Institute of Technology, Cambridge, MA USA; 7 Klarman Cell Observatory, Broad Institute of MIT and Harvard, Cambridge, MA USA; 8 Department of Chemistry and Chemical Biology, Harvard University, Cambridge, MA USA; 9 Stanley Center for Psychiatric Research, Broad Institute of MIT and Harvard, Cambridge, MA USA

**Keywords:** RNA sequencing, Molecular neuroscience, Transcriptomics, Transcriptomics, Genetic vectors

Correction to: *Nature* 10.1038/s41586-023-06569-5 Published online 27 September 2023

This article was originally published under a standard Springer Nature licence (© The Author(s), under exclusive licence to Springer Nature Limited). It is now available as an open-access paper under a Creative Commons Attribution 4.0 International licence, © The Author(s). The change has been made in the HTML and PDF versions of the article.

