# Supplementary material for: Spatial atlas of the mouse central nervous system at molecular resolution
Source: Nature. 2023 Sep 27;622(7983):552–61. doi: 10.1038/s41586-023-06569-5 (PMC10709140; doi:10.1038/s41586-023-06569-5)
Supplement: Supplementary file 1 — Supplementary Information [file 41586_2023_6569_MOESM1_ESM.pdf]

nature portfolio

https://doi.org/10.1038/s41586-023-06569-5

Supplementary information

Spatial atlas of the mouse central nervous system at molecular resolution

In the format provided by the authors and unedited

Supplementary Information

Spatial Atlas of the Mouse Central Nervous System at Molecular Resolution

Hailing Shi[1,2,10], Yichun He[1,3,10], Yiming Zhou[1,2,10], Jiahao Huang[1,2], Kamal Maher[1,4], Brandon Wang[1,5,6], Zefang Tang[1,2], Shuchen Luo[1,2], Peng Tan[1,7], Morgan Wu[1], Zuwan Lin[1,3,8], Jingyi Ren[1,2], Yaman Thapa[1], Xin Tang[1,3], Ken Y. Chan[1,9], Benjamin E. Deverman[1,9], Hao Shen[3], Albert Liu[1,2], Jia Liu[3*] & Xiao Wang[1,2,9*]

[1] Broad Institute of MIT and Harvard, Cambridge, MA, USA.
[2] Department of Chemistry, Massachusetts Institute of Technology, Cambridge, MA, USA.
[3] John A. Paulson School of Engineering and Applied Sciences, Harvard University, Boston, MA, USA.
[4] Computational and Systems Biology Ph.D. Program, Massachusetts Institute of Technology, Cambridge, MA, USA.
[5] Department of Electrical Engineering and Computer Science, Massachusetts Institute of Technology, Cambridge, MA, USA.
[6] Department of Biology, Massachusetts Institute of Technology, Cambridge, MA, USA.
[7] Klarman Cell Observatory, Broad Institute of MIT and Harvard, Cambridge, MA, USA.
[8] Department of Chemistry and Chemical Biology, Harvard University, Cambridge, MA, USA.
[9] Stanley Center for Psychiatric Research, Broad Institute of MIT and Harvard, Cambridge, MA, USA.
[10] These authors contributed equally to this work: Hailing Shi, Yichun He, and Yiming Zhou
[*] Correspondence e-mail: xwangx@mit.edu; jia_liu@seas.harvard.edu

Contents

Supplementary Discussion **2**
- Sample preparation and damage evaluation 2
- Imputation performance and evaluation 2

Supplementary Notes **3**
- Tissue region abbreviations 3

Supplementary Figures **5**
- Supplementary Figure 1 5
- Supplementary Figure 2 6
- Supplementary Figure 3 8
- Supplementary Figure 4 9

Legends for Supplementary Tables **10**

Supplementary References **10**

Supplementary Discussion

Sample preparation and damage evaluation

STARmap PLUS tissue collection

During STARmap PLUS tissue sample collection, the whole mouse brain was freshly collected shortly after rapid decapitation (< 5 min), embedded in OCT, flash-frozen in liquid nitrogen (~ 10 minutes), and kept at -80 ^{o}C until brain slice sectioning (Supplementary Fig. 1a). The brain tissues were sectioned at -20 ^{o}C with a cryostat, adhered to a coverslip, and immediately fixed with 4% paraformaldehyde (PFA) in PBS. The tissue samples were processed in frozen format until PFA fixation to minimize disturbance to the tissue and degradation of RNA, which can be reflected by the lower percentage of activated microglia in the whole microglia population (*Ccl3*$^{+}$ or *Ccl4*$^{+}$, 8.8% in the current atlas versus 24.6% in the scRNA-seq atlas[1]).

Tissue sectioning may result in cell fragments at the slice surface. However, the STARmap PLUS and ClusterMap methods include three following steps of quality control to address this issue: (i) small cell fragments without clear nuclear DAPI staining were filtered out; (ii) small cell fragments containing fewer than 30 reads or fewer than 20 genes were further filtered out; and (iii) variation brought by cell volume is normalized by counts per cell during pre-processing before cell clustering.

Cell clusters quality check

We compared the number of reads and number of genes among subclusters (Supplementary Fig. 1b-d). First, we observed a high correlation between the median genes per cell and the median reads per cell among subclusters (Supplementary Fig. 1b), indicating consistent detection efficiency among genes. Furthermore, there was no correlation between the cluster size (whether in terms of the number of cells in the subcluster, Supplementary Fig. 1c; or the subcluster's population percentage within its main cluster, Supplementary Fig. 1d) and the number of reads per cell or the number of genes per cell, thereby ruling out the possibility that small clusters were a result of low-quality cells caused by tissue damage or RNA degradation during sample preparation.

Imputation performance and evaluation

Gene expression features associated with imputation performance

Using the genes with STARmap PLUS measured ground-truth, we examined the following four gene expression features for their association with the imputation performance score in the "leave-one-(gene)-out" intermediate imputation (Supplementary Fig. 4).

(1) Gene expression level in STARmap PLUS. Genes were categorized into four groups based on total read count in the STARmap PLUS dataset. Imputation performance shows an increasing trend as gene expression level increases (Supplementary Fig. 4a; Pearson's r = 0.443, P = 4.6e-50).

(2) Spatial expression heterogeneity in STARmap PLUS. For each gene, Moran's *I* (a coefficient measuring overall spatial autocorrelation[51]) for the gene's spatial expression was calculated for each of the 20 sample slices and then averaged, to represent the degree of patterned spatial expression. A higher Moran's *I* represents more patterned spatial gene expression. We observed a positive correlation between the spatial pattern and imputation performance (Supplementary Fig. 4b, Pearson's r = 0.738, P = 2.3e-175).

(3) Gene expression in scRNA-seq dataset[1]. Similar to (1), we observed higher imputation performance for genes with higher read counts in the scRNA-seq dataset[1] (Supplementary Fig. 4c, Pearson's r = 0.209, P = 1.7e-11).

(4) Single-cell expression heterogeneity in scRNA-seq dataset[1]. We quantified the degree of cell expression specificity of a gene by calculating Moran's *I* of the scRNA-seq[1] UMAP plot coloured by the gene's expression. Genes with a higher Moran's *I* on UMAP (usually cell cluster marker genes) tend to have better imputation performance (Supplementary Fig. 4d, Pearson's r = 0.517, P = 1.3e-70).

We observed that gene expression heterogeneity in space and in single cells has a greater impact on imputation performances compared to gene expression levels (Supplementary Fig. 4), and genes with expression heterogeneity tend to have better imputation performance (Extended Data Fig. 9b). These observations were consistent with a recent spatial expression gene imputation report[70], which showed that cell type-specific expressed genes and more highly expressed genes exhibit higher prediction accuracy. A gene's cell-type specificity (e.g., examining single-cell expression profiles in an atlas), spatial distribution (e.g., referencing Allen *In Situ* Hybridization database[23]) and expression level can be important considerations when evaluating and judging gene imputation results.

Supplementary Notes

Tissue region abbreviations

Abbreviations of tissue regions were labeled based on the Allen Mouse Brain Reference Atlas[18-20]. Tissue region abbreviations: ACA, anterior cingulate area; AI, agranular insular area; AIp, agranular insular area, posterior part; alv, alveus; AOBgr, accessory olfactory bulb, granule layer; AON, anterior olfactory nucleus; AONm, anterior olfactory nucleus, medial part; AQ, cerebral aqueduct; ARH, arcuate hypothalamic nucleus; AUD, auditory area; BLA, basolateral amygdalar nucleus; BMA, basomedial amygdalar nucleus; BSTv, bed nuclei of the stria terminalis anterior division ventral nucleus; BSTd, bed nuclei of the stria terminalis posterior division dorsal nucleus; CA1sp, field CA1, pyramidal layer; CA2sp, field CA2, pyramidal layer; CA3sp, field CA3, pyramidal layer; CBXd-gr, cerebellar cortex, dorsal part, granular layer; CBXmo, cerebellar cortex, molecular layer; CBXpu, cerebellar cortex, Purkinje layer; CBXv-gr, cerebellar cortex, ventral part, granular layer; cc, corpus callosum; CEA, central amygdalar nucleus; CEAl, central amygdalar nucleus, lateral part; chpl, choroid plexus; cing, cingulum bundle; CLA, claustrum; COAa, cortical amygdalar area, anterior part; COAp, cortical amygdalar area, posterior part; CP, caudoputamen; CTX, cerebral cortex; CTXsp, cortical subplate; df, dorsal fornix; DG, dentate gyrus; DGd-sg,

dentate gyrus, dorsal part, granule cell layer; DGmo/po, dentate gyrus, molecular layer/polymorph layer; DGv-sg, dentate gyrus, ventral part, granule cell layer; DMH, dorsomedial nucleus of the hypothalamus; DMX, dorsal motor nucleus of the vagus nerve; DR, dorsal nucleus raphe; ECT, ectorhinal area; ENTl, entorhinal area, lateral part; ENTm, entorhinal area, medial part; EP, endopiriform nucleus; EW, Edinger-Westphal nucleus; FB, forebrain; Fbr, fiber tracts; FC, fasciola cinerea; fi, fimbria; HB, hindbrain; HBl, hindbrain lateral part; HPF, hippocampal formation; HPFslm/sr/so, hippocampal formation stratum lacunosum-moleculare/stratum radiatum/stratum oriens; HYal, hypothalamus, anterior-lateral enriched; HYam, hypothalamus, anterior medial enriched; HYpm, hypothalamus, posterior-medial part enriched; IA, intercalated amygdalar nucleus; IC, inferior colliculus; IG, indusium griseum; III, oculomotor nucleus; ILA, infralimbic area; int, internal capsule; IO, inferior olivary complex; IPN, interpeduncular nucleus; L1l, cerebral cortical layer 1, lateral part; L1m, cerebral cortical layer 1, medial part; L2/3, layer 2/3; L4, layer 4; L5, layer 5; L6, layer 6; L6a, layer 6a; L6b, layer 6b; LA, lateral amygdalar nucleus; LC, locus coeruleus; LDT, laterodorsal tegmental nucleus; LH, lateral habenula; LHA, lateral hypothalamic area; LS, lateral septal nucleus; MEA, medial amygdalar nucleus; MH, medial habenula; MHd, medial habenula, dorsal part; MHv, medial habenula, ventral part; MMa, medial mammillary nucleus, anterior part; MMp, medial mammillary nucleus, posterior part; MNG, meninges; mo, molecular layer; MO, somatomotor areas; MOp, primary motor area; MOBgr, main olfactory bulb, granule layer; mPFC, medial prefrontal cortex; MYa, medulla, anterior enriched; MYd, medulla, dorsal part; MYm, medulla, medial enriched; MYp, medulla, posterior enriched; MV, medial vestibular nucleus; NTS, nucleus of the solitary tract; OBgl, olfactory bulb, glomerular layer; OBmi, olfactory bulb, mitral layer; OBopl, olfactory bulb, outer plexiform layer; onl, olfactory nerve layer of main olfactory bulb; P, pons; PA, posterior amygdalar nucleus; PALd, pallidum, dorsal region; PALm, pallidum, medial region; PALv, pallidum, ventral region; PAGd, periaqueductal gray, dorsal part enriched; PAGpv, periaqueductal gray, posterior ventral part; PCG, pontine central gray; PERI, perirhinal area; PIR, piriform area; PL, prelimbic area; Pm, pons, medial part; PPN, pedunculopontine nucleus; po, polymorph layer; POST, postsubiculum; PRE, presubiculum; PSV, principal sensory nucleus of the trigeminal; PVH, paraventricular hypothalamic nucleus; PVp, periventricular hypothalamic nucleus, posterior part; RE, nucleus of reuniens; RO, nucleus raphe obscurus; RPA, nucleus raphe pallidus; RR, midbrain reticular nucleus, retrorubral area; RSP, retrosplenial cortex; RT, reticular nucleus of the thalamus; sAMY, striatum-like amygdalar nuclei; SC, superior colliculus; SCH, suprachiasmatic nucleus; SCO, subcommissural organ; SEZ, subependymal zone; SFO, subfornical organ; sg, granule cell layer; slm, stratum lacunosum-moleculare; SNc, substantia nigra, compact part; SNr, substantia nigra, reticular part; so, stratum oriens; sp, pyramidal layer; SPV, spinal nucleus of the trigeminal; sr, stratum radiatum; SS, somatosensory area; SSp, primary SS; SSs, secondary SS; STN, subthalamus nucleus; STR, striatum; STR-periV, periventricular area of striatum; STRd-al, dorsal striatum, anterior-lateral enriched; STRd-pm, dorsal striatum, posterior-medial enriched; STRv, striatum ventral region; STRv-al, ventral striatum, anterior-lateral enriched; STRv-isl, ventral striatum, islands of Calleja; STRv-OT, ventral striatum, olfactory tubercle; STRv-pm, ventral striatum, posterior-medial enriched; SUBsp, subiculum, pyramidal layer; SUBsr, subiculum stratum radiatum; TEa, temporal association area; TH, thalamus; THl, lateral TH; THam, anterior-medial TH; THm, thalamus medial part; THpm, posterior medial TH; TM, tuberomammillary nucleus; TRS, triangular nucleus of septum; TTd, taenia tecta, dorsal part; TTv, taenia tecta, ventral part; V, motor nucleus of

trigeminal; v3, third ventricle; VII, facial motor nucleus; VIS, visual area; VISC, visceral area; VL, lateral ventricle; VMH, ventromedial hypothalamic nucleus; VTA, ventral tegmental area; VW, ventricular wall; ZI, zona incerta.

Supplementary Figures

Supplementary Figure 1

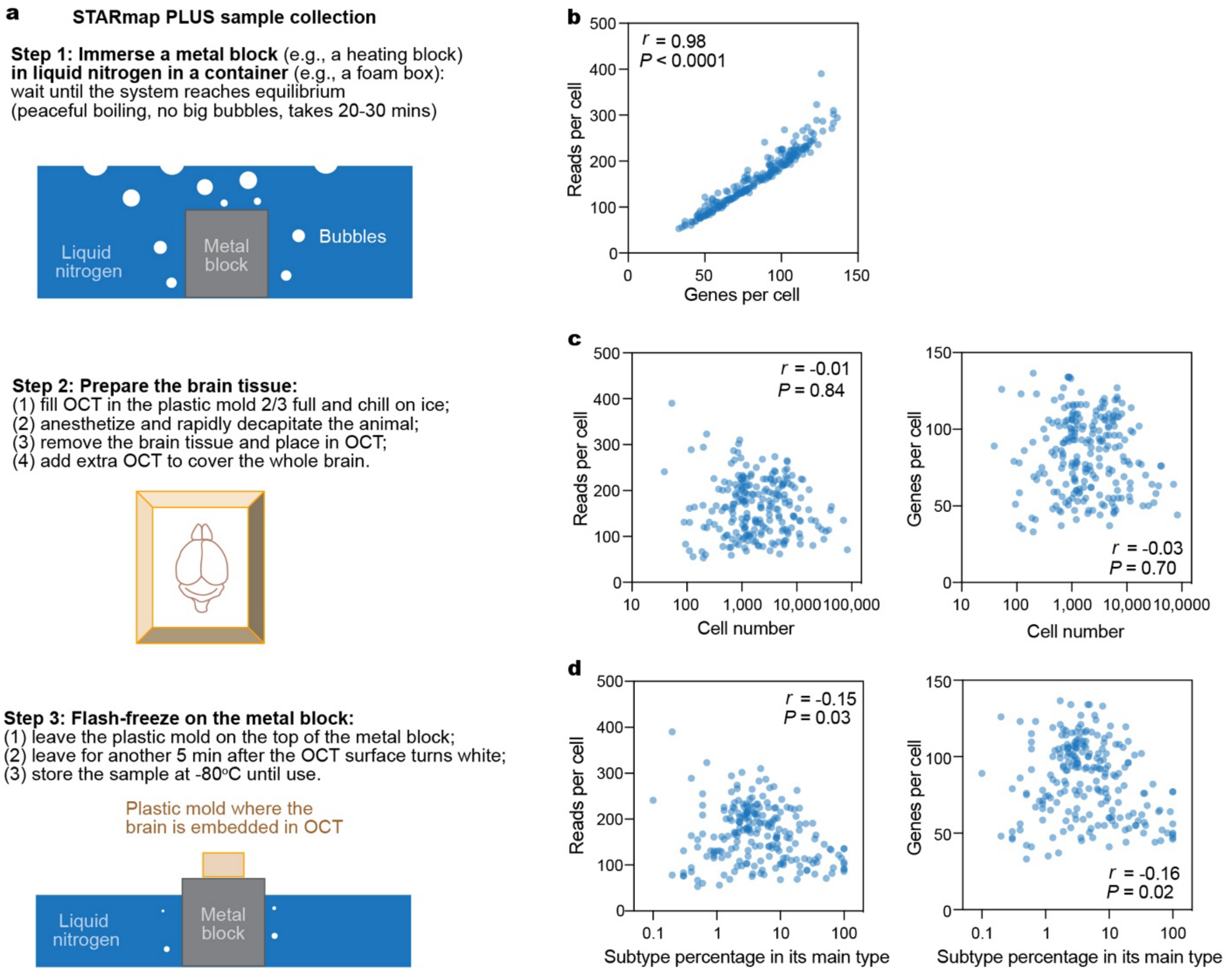

Supplementary Figure 1 | STARmap PLUS sample collection and quality controls of cell clusters. a, Schematics of brain tissue collection in STARmap PLUS. The brain was quickly removed from the sacrificed animal and flash-frozen by liquid nitrogen to minimize disturbing tissue and RNA quality. **b**, Scatter plot of the number of genes per cell versus the number of reads per cell in subclusters. *n* = 230. **c,d**, Scatter plots of the subcluster size (**c**, *n* = 230) or subcluster population percentage in the main cluster (**d**, *n* = 218, NA subclusters not included) versus the number of reads per cell (left) or the number of genes per cell (right). Each dot represents a cell subcluster; the median value of the cluster was plotted (**b-d**). Spearman's *r* and *P* values (two-tailed) were calculated with GraphPad Prism Version 9.3.1. (**b-d**). Data are provided in the accompanying Source Data file.

Supplementary Figure 2

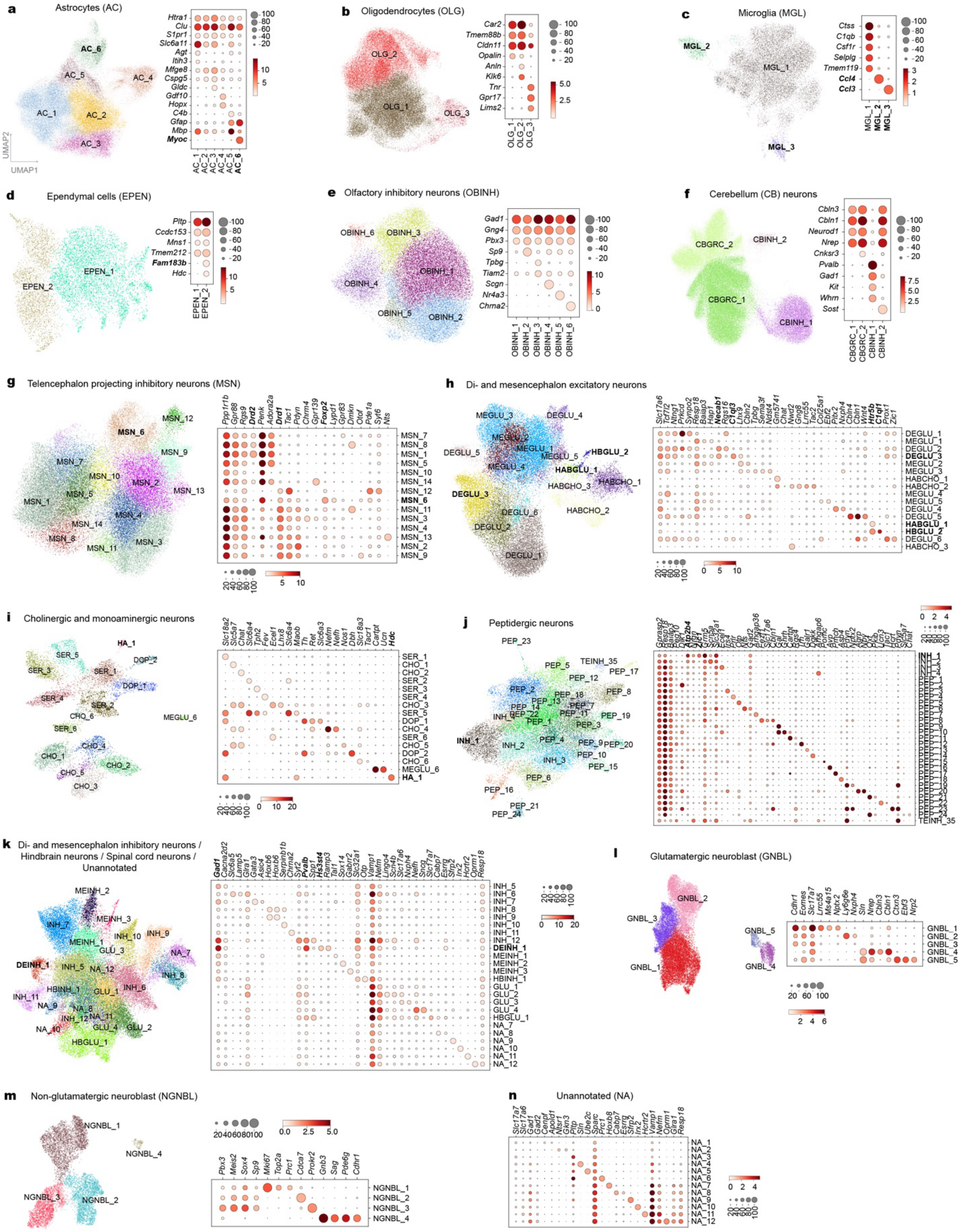

Supplementary Figure 2 | Subclustering of main cell types. Uniform Manifold Approximation and Projection (UMAP) maps (left) and marker gene dot plots (right) of main clusters coloured by

cell subcluster identities, for astrocytes (AC, **a**), oligodendrocytes (OLG, **b**), microglia (MGL, **c**), ependymal cells (EPEN, **d**), olfactory inhibitory neurons (OBINH, **e**), cerebellum (CB) neurons (**f**), telencephalon projecting inhibitory neurons (MSN, **g**), di- and mesencephalon excitatory neurons (**h**), cholinergic and monoaminergic neurons (**i**), peptidergic neurons (**j**), di- and mesencephalon inhibitory neurons/hindbrain neurons/spinal neurons/unannotated (**k**), glutamatergic neuroblasts (**l**), and non-glutamatergic neuroblasts (**m**). **n**, Marker gene dot plot for unannotated (NA) clusters. Dot sizes, the fraction of cells in the group; color bars, mean expression level in the group. Cell types and genes mentioned in the main text are bolded. Also see Methods and Supplementary Table 4.

Supplementary Figure 3

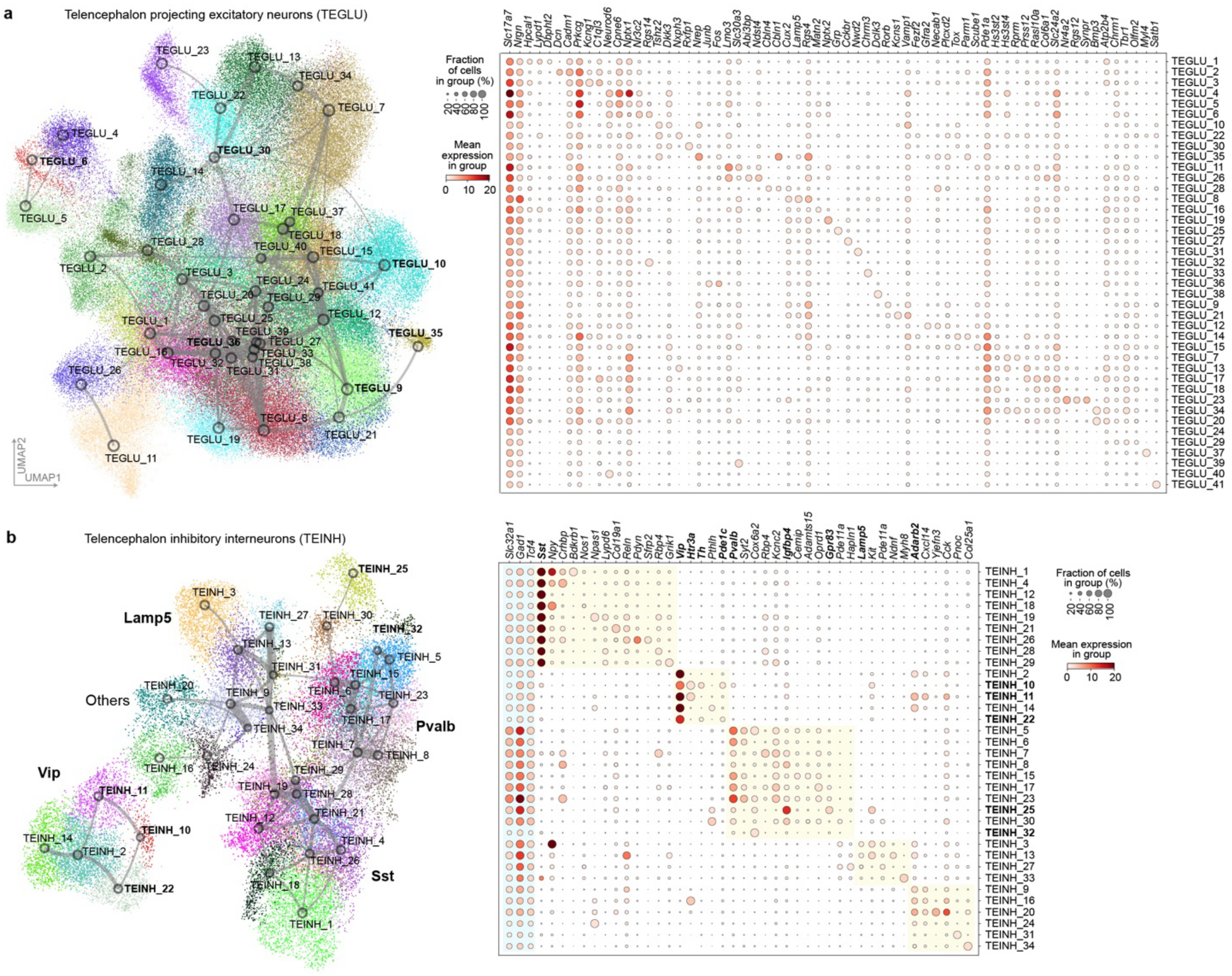

Supplementary Figure 3 | Subclustering of telencephalon projecting excitatory neurons and telencephalon inhibitory interneurons. a,**b**, Overlapped UMAP and constellation plot[26] of main clusters coloured by cell subcluster identities (left) and marker gene dot plots (right), for telencephalon projecting excitatory neurons (TEGLU, **a**) and telencephalon inhibitory interneurons (TEINH, **b**). Also see Methods and Supplementary Table 4.

Supplementary Figure 4

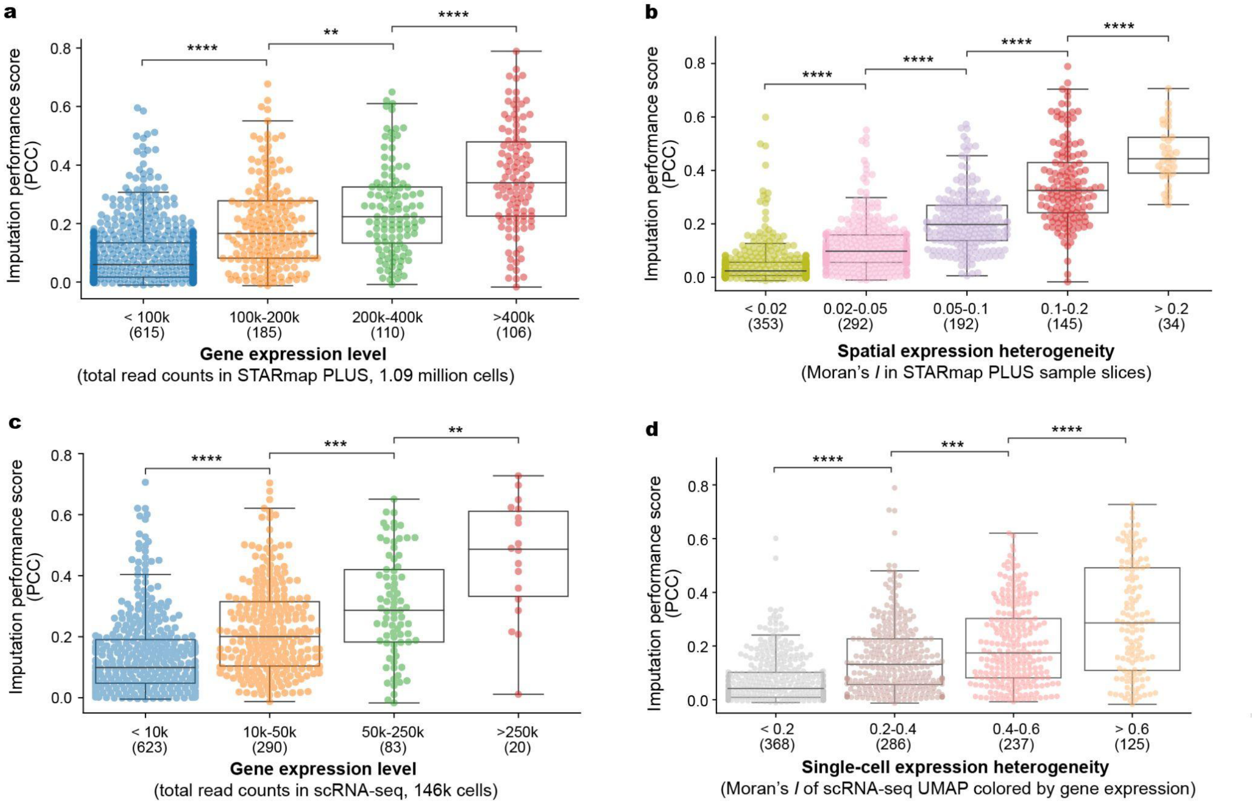

Supplementary Figure 4 | Imputation performance and gene expression features. a-d, Box plots of imputation performance scores of genes of various expression features. Genes were divided into multiple groups based on their expression level in STARmap PLUS (**a**), spatial expression heterogeneity (**b**), expression level in the scRNA-seq atlas[1] (**c**), or single-cell expression heterogeneity in the scRNA-seq atlas[1] (**d**). PCC, Pearson's correlation coefficient between a gene's imputed values and measured STARmap PLUS expression level across cells. *P* values were calculated with two-sided Mann-Whitney-Wilcoxon tests. ***P* < 0.01, ****P* < 0.001, *****P* < 0.0001. Boxplot elements: the vertical line, median; the box, first to third quartiles; whiskers, smallest and largest data points excluding outliers. Numbers in parentheses, number of genes. Also see Supplementary Table 7 and Supplementary Discussion.

Legends for Supplementary Tables

Supplementary Table 1: 1,022 gene list of STARmap PLUS and the 5-nt code for each gene.
Supplementary Table 2: Sequences of probes used in this study, including SNAIL probes (tab 1 for endogenous genes; tab 2 for RNA barcodes), SEDAL probes (tab 3), and smFISH-HCR probes (tab 4).
Supplementary Table 3: Detailed information for mice and tissue slices in the atlas (tab 1) and read count in each tissue slice (tab 2).
Supplementary Table 4: Molecular cell subcluster annotation, including cell type symbols, cell numbers, colour codes, cell type description, cell states, cross-dataset correspondence (tab 1); Cell-cell adjacency quantification (tab 2: among main cell types; tab 3: among subcluster cell types).
Supplementary Table 5: Molecular tissue region annotation, including tissue region symbol, description, marker genes (tab 1: top-level; tab 2: sublevel); and molecular cell-type composition (tab 3); Parameters of neighbor size and clustering resolution in molecular tissue region clustering (tab 4).
Supplementary Table 6: Molecular spatial cell clusters (cluster size > 50).
Supplementary Table 7: Intermediate mapping performance evaluation (tab 1), imputed mean gene expression in sublevel tissue regions in sample slice 12 (tab 2), and predicted marker genes in the dorsal (tab 3) and ventral (tab 4) medial habenula.
Supplementary Table 8: AAV-PHP.eB transduction quantification across 106 sublevel molecular tissue regions (tabs 1&2), 26 main cell types (tabs 3&4), and 230 subtypes (tabs 5&6).

Supplementary References

70. Zhang, C., Chen, R. & Zhang, Y. Accurate inference of genome-wide spatial expression with iSpatial. *Sci Adv* **8**, eabq0990 (2022).
