# Supplementary material for: Spatial atlas of the mouse central nervous system at molecular resolution
Source: Nature. 2023 Sep 27;622(7983):552–61. doi: 10.1038/s41586-023-06569-5 (PMC10709140; doi:10.1038/s41586-023-06569-5)
Supplement: Supplementary file 2 — Reporting Summary [file 41586_2023_6569_MOESM2_ESM.pdf]

nature portfolio

Corresponding author(s): Xiao WANG, Jia LIU

Last updated by author(s): Sep 9, 2023

Reporting Summary

Nature Portfolio wishes to improve the reproducibility of the work that we publish. This form provides structure for consistency and transparency in reporting. For further information on Nature Portfolio policies, see our Editorial Policies and the Editorial Policy Checklist.

Statistics

For all statistical analyses, confirm that the following items are present in the figure legend, table legend, main text, or Methods section.

n/a	Confirmed	
☐	☒	The exact sample size (*n*) for each experimental group/condition, given as a discrete number and unit of measurement
☐	☒	A statement on whether measurements were taken from distinct samples or whether the same sample was measured repeatedly
☐	☒	The statistical test(s) used AND whether they are one- or two-sided<br>*Only common tests should be described solely by name; describe more complex techniques in the Methods section.*
☒	☐	A description of all covariates tested
☐	☒	A description of any assumptions or corrections, such as tests of normality and adjustment for multiple comparisons
☐	☒	A full description of the statistical parameters including central tendency (e.g. means) or other basic estimates (e.g. regression coefficient) AND variation (e.g. standard deviation) or associated estimates of uncertainty (e.g. confidence intervals)
☐	☒	For null hypothesis testing, the test statistic (e.g. *F*, *t*, *r*) with confidence intervals, effect sizes, degrees of freedom and *P* value noted<br>*Give P values as exact values whenever suitable.*
☒	☐	For Bayesian analysis, information on the choice of priors and Markov chain Monte Carlo settings
☒	☐	For hierarchical and complex designs, identification of the appropriate level for tests and full reporting of outcomes
☐	☒	Estimates of effect sizes (e.g. Cohen's *d*, Pearson's *r*), indicating how they were calculated

Our web collection on statistics for biologists contains articles on many of the points above.

Software and code

Policy information about availability of computer code

Data collection	STARmap PLUS images were acquired using Leica TCS SP8 or Stellaris 8 confocal microscopy with Leica LAS X software (SP8: version 3.5.5.19976; Stellaris 8: version 4.4.0.24861).
Data analysis	Image deconvolution was achieved with Huygens Essential version 21.04 (Scientific Volume Imaging, The Netherlands, http://svi.nl), using the Classic Maximum Likelihood Estimation (CMLE) method, with SNR:10 and 10 iterations. Image registration, spot calling, and barcode filtering were applied according to previous reports (Wang X. et al, Science, 361(2018); Zeng H. et al, bioRxiv (2022), doi:10.1101/2022.01.14.476072). ClusterMap is implemented based on MATLAB R2019b and Python 3.6. The following packages and software were used in data analysis: UCSF ChimeraX 1.0, ImageJ 1.51, MATLAB R2019b, R 4.0.4, RStudio 1.4.1106, Jupyter Notebook 6.0.3, Anaconda 2-2-.02, h5py 3.1.0, hdbscan 0.8.36, hdf5 1.10.4, matplotlib 3.1.3, seaborn 0.11.0, scanpy 1.6.0, numpy 1.19.4, scipy 1.6.3, pandas 1.2.3, scikit-learn 0.22, umap-learn 0.4.3, pip 21.0.1, numba 0.51.2, tifffile 2020.10.1, scikit-image 0.18.1, itertools 8.0.0, Squidpy 1.1.2, anndata 0.8.0. The code that supports the analyses in this study is available at https://github.com/wanglab-broad/mCNS-atlas.<br>Spearman's r and its P values (two-tailed) in Supplementary Fig. 1 and Pearson's r and its P values (two-tailed) in Supplementary Discussion were calculated with GraphPad Prism Version 9.3.1. P values in Supplementary Fig. 4 were calculated with two-sided Mann-Whitney-Wilcoxon tests by statannotations (version 0.4.4) using the function statannotations.Annotator.annotator.configure(test='Mann-Whitney', text_format='star', loc='outside'). **P < 0.01, ***P < 0.001, ****P < 0.0001.

For manuscripts utilizing custom algorithms or software that are central to the research but not yet described in published literature, software must be made available to editors and reviewers. We strongly encourage code deposition in a community repository (e.g. GitHub). See the Nature Portfolio guidelines for submitting code & software for further information.

Data

Policy information about availability of data

All manuscripts must include a data availability statement. This statement should provide the following information, where applicable:

- Accession codes, unique identifiers, or web links for publicly available datasets
- A description of any restrictions on data availability
- For clinical datasets or third party data, please ensure that the statement adheres to our policy

The STARmap PLUS sequencing data of this study are available on the Single Cell Portal (https://singlecell.broadinstitute.org/single_cell/study/SCP1830) and Zenodo (https://doi.org/10.5281/zenodo.8327576). We also introduced an interactive online database (http://brain.spatial-atlas.net) for exploratory analysis and hypothesis generation. Source data for figures are provided with this paper. Publicly used databases in the study: Allen Mouse Brain Reference Atlas (In situ hybridization, CCFv3); single-cell RNA-seq datasets of adult mouse nervous system (available at Sequence Read Archive (https://www.ncbi.nlm.nih.gov/sra) under accession SRP135960), cerebellum (available at Gene Expression Omnibus (GEO) under accession number GSE165371), striatum (available at GEO under accession number GSE118020), and whole cortex and hippocampus (in the NeMO Archive for the BRAIN Initiative Cell Census Network, https://assets.nemoarchive.org/dat-jb2f34y); and processed AAV-PHP.eB transduction rate in cortical cell types (available at CaltechData, http://dx.doi.org/10.22002/D1.2090, “aavomics_cell_type_transduction_rates.csv”).

Field-specific reporting

Please select the one below that is the best fit for your research. If you are not sure, read the appropriate sections before making your selection.

☒ Life sciences ☐ Behavioural & social sciences ☐ Ecological, evolutionary & environmental sciences

For a reference copy of the document with all sections, see nature.com/documents/nr-reporting-summary-flat.pdf

Life sciences study design

All studies must disclose on these points even when the disclosure is negative.

Sample size	For 1,022-gene mapping experiments, 20 tissue slices across the mouse central nervous system from three mice (two female, one male) were used (Supplementary Table 3). No statistical methods were used to predetermine sample sizes. The 20 tissue slices have sufficient coverage of major brain structures and yielded a cell number comparable to previous brain-wide single-cell profiling publications.
Data exclusions	Quality control steps were performed to filter low-quality signals, including (1) barcode filtering in the spot-calling step; (2) pre-processing in the ClusterMap pipeline to filter out local low-density mRNA spots; (3) post-processing in the ClusterMap pipeline to filter out 'cells' that do not overlap with DAPI signals; (4) standard preprocessing procedures in Scanpy to filter low-quality cells.<br>Details of analysis are in Methods part.
Replication	1. STARmap PLUS measurements were not directly replicated. However, the data were generated from three animals (2 female and 1 male) and pooled together for analysis.<br>2. Two independent biological replicates of AAV tropism quantification experiments were performed. Replicate 1, 17 coronal tissue slices from two mice; Replicate 2, 3 sagittal tissue slices from one mouse. The AAV tropism profiles were reproducible at the tissue-region and cell-type levels in the two replicates (Extended Data Figure 10 and Supplementary Table 8).<br>3. smFISH-HCR (single-molecule fluorescence in situ hybridization with hybridization chain reaction) were performed with two to three sample slices from one or two animals.
Randomization	There was no randomization performed as the study does not involve multiple study groups. STARmap PLUS datasets for the 20 tissue slices were collected in multiple batches yet with the standard protocols in library preparation and imaging acquisition. A batch correction step was performed when necessary when compiling data from all tissue slices (Supplementary Table 3 and Methods).
Blinding	There was no blinding performed as the study does not involve multiple study groups. During the batch correction, knowledge of the tissue slide identity was necessary for analytical pipelines to group relevant samples. During data integration, knowledge of the cell source (single-cell RNA-sequencing OR STARmap PLUS) was necessary for analytical pipelines to evaluate integration performance. Clustering of single-cell transcriptomes then was performed blind to the mouse source or any other metadata that could reveal sample identity.

Reporting for specific materials, systems and methods

We require information from authors about some types of materials, experimental systems and methods used in many studies. Here, indicate whether each material, system or method listed is relevant to your study. If you are not sure if a list item applies to your research, read the appropriate section before selecting a response.

Materials & experimental systems

n/a	Involved in the study	
☒	☐	Antibodies
☐	☒	Eukaryotic cell lines
☒	☐	Palaeontology and archaeology
☐	☒	Animals and other organisms
☒	☐	Human research participants
☒	☐	Clinical data
☒	☐	Dual use research of concern

Methods

n/a	Involved in the study	
☒	☐	ChIP-seq
☒	☐	Flow cytometry
☒	☐	MRI-based neuroimaging

Eukaryotic cell lines

Policy information about cell lines

Cell line source(s)	HEK 293T cells (ATCC® CRL-3216™)
Authentication	The cell line was authenticated by the vendor using tests/methods including passage number, post-freeze viability, growth properties, morphology, mycoplasma contamination test, species determination, sterility test, and human pathogenic virus testing.
Mycoplasma contamination	Negative. The cell line was tested negative for mycoplasma contamination by ATCC. During maintenance, the cell line is regularly tested with Hoechst DNA stain method.
Commonly misidentified lines (See ICLAC register)	None.

Animals and other organisms

Policy information about studies involving animals; ARRIVE guidelines recommended for reporting animal research

Laboratory animals	C57BL/6 (000664, female, 8-10 weeks old; male, 10-13 weeks old) and B6.Cg-Tg(Thy1-YFP)HJrs/J (003782, male, 5 weeks old). Tissues were collected 4-5 weeks after AAV administration. Animals were housed 2-5 per cage and kept on a 12-hour light-dark cycle with ad libitum food and water.
Wild animals	None.
Field-collected samples	None.
Ethics oversight	Experimental procedures were approved by the Institutional Animal Care and Use Committee (IACUC) of the Broad Institute of MIT and Harvard under animal protocol # 0255-08-19.

Note that full information on the approval of the study protocol must also be provided in the manuscript.
